# Stable Na Deposition/Dissolution Enabled by 3D Bimetallic Carbon Fibers with Artificial Solid Electrolyte Interface

**DOI:** 10.1002/smsc.202400655

**Published:** 2025-04-29

**Authors:** Sandro Schöner, Dana Schmidt, Leonie Wildersinn, Stephanie E. Wolf, Sebastian Speer, Beatrice Wolff, Arseniy Bokov, Pengfei Cao, Anna Windmüller, Xiaoxuan Chen, Chih‐Long Tsai, Fabian Jeschull, Hermann Tempel, Shicheng Yu, Rüdiger‐A. Eichel

**Affiliations:** ^1^ Institute of Energy Technologies – Fundamental Electrochemistry (IET‐1) Forschungszentrum Jülich 52428 Jülich Germany; ^2^ Material and Processes of Electrochemical Energy Storage and Conversion RWTH Aachen University 52074 Aachen Germany; ^3^ Karlsruher Institute of Technologie (KIT) Institute for Applied Materials‐Energy Storage Systems (IAM‐ESS) 76344 Eggenstein‐Leopoldshafen Germany; ^4^ Helmholtz Institute Ulm (HIU) Electrochemical Energy Storage (EES) 89081 Ulm Germany; ^5^ Ernst Ruska‐Centre for Microscopy and Spectroscopy with Electrons Forschungszentrum Jülich 52428 Jülich Germany; ^6^ Institute of Energy Materials and Devices ‐ Helmholtz Institute Münster: Ionics in Energy Storage (IMD‐4/HI MS) Forschungszentrum Jülich 48149 Münster Germany

**Keywords:** 3D interlayer, artificial solid electrolyte interfaces, Na depositions, presodiation, sodiophilic–sodiophobic gradients

## Abstract

3D bimetallic carbon nanofibers (CNFs) are promising interlayers for regulating Na deposition/dissolution on the Na metal or directly on current collectors like Cu. However, uncontrollable solid electrolyte interface (SEI) growth on the interlayer during the repeated Na plating/stripping process leads to low initial Coulombic efficiency (CE), impeding the practical applications of such a protective layer in Na metal batteries. Herein, an artificial SEI‐coated interlayer decorated with sodiophilic Ag and sodiophobic Cu on CNF is applied on Cu foil to regulate the Na deposition/dissolution behavior. The artificial SEI, consisting of organic components like RCO_2_Na/RCONa and inorganic reactants Na_2_CO_3_/Na_x_O_y_, minimizes irreversible electrolyte decomposition at the interlayer. The sodiophobic–sodiophilic bimetallic CNF interlayer is lightweight, porous, and mechanically robust. It can guide Na deposition toward the sodiophilic Ag‐rich region of the CNF matrix and cluster in the open pores facing the current collector, effectively preventing Na dendrite formation. The interlayer features with artificial SEI synergistically enhance the stability of Na deposition/dissolution on Cu foil, resulting in a high average CE of over 99.5% for 600 cycles spanning 6500 h. Furthermore, post‐analysis confirms the high electrochemical stability of the artificial SEI of the interlayer during cycling.

## Graphical abstract

The combination of three‐dimensional bimetallic carbon fibers with an artificial SEI created through chemical presodiation enables a stable and homogeneous sodium deposition/dissolution process.

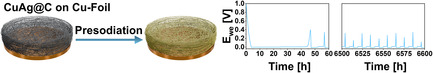

## 1 Introduction

Metallic Na is a very abundant metal with a high gravimetric capacity of 1166 mA h g^−1^, a low redox potential of −2.714 V versus the standard hydrogen electrode. Therefore, it is considered a promising alternative to Li electrodes for post‐Li‐ion batteries, such as anode‐less Na batteries, where the Na metal electrode is deposited directly on a current collector.^[^ ^1^ ^]^ However, depositing Na metal on a current collector like sodiophobic Cu still presents significant challenges in practical applications. During cycling, issues such as substantial volume expansion of Na and uneven Na deposition/dissolution can lead to Na dendrite formation and active Na loss, ultimately reducing Coulombic efficiency (CE), shortening cycle life, and posing potential safety risks.^[^ ^2^, ^3^, ^4^, ^5^ ^]^

Introducing a 3D porous CNF based interlayer can alleviate the volume expansion problem by reducing the built‐in stress during sodium deposition and thus minimizing volume changes.^[^ ^6^, ^7^, ^8^ ^]^ Additionally, such CNF‐based interlayers can be decorated with various sodiophobic and/or sodiophilic metal particles. Sodiophilic substrates, such as Au, Ag, and Sn, lower the nucleation overpotential for Na deposition. The negative Gibbs formation energy between the substrate and sodium ensures that sodium nucleation first occurs at these sodiophilic substrates, promoting homogeneous deposition.^[^ ^9^ ^]^ In contrast, sodiophobic substances such as Cu or Mo are energetically less favored for Na nucleation, preventing concentrated sodium deposition on the “top” side facing the separator and uncontrollable Na agglomerate growth in the interlayer.^[^ ^10^, ^11^ ^]^ Combining both sodiophobic and sodiophilic effects enables uniform Na deposition and significantly enhances the stability of the plating/stripping process.^[^ ^12^ ^]^

The formation and stabilization of the solid electrolyte interface (SEI) on 3D porous interlayers, particularly for carbon‐based materials, present significant challenges. Due to the increased surface area resulting from high porosity and the defects in the carbon structure, a substantial amount of Na‐ions are consumed during the initial formation of the SEI in the first cycle, leading to a low initial CE.^[^ ^13^ ^]^ This consumption occurs because SEIs form via decomposition reactions between electrolyte components and Na‐ions at the surface of the negative electrode.

The decomposed organic and inorganic products accumulate on the surface of the interlayer, creating an SEI, which can cause a rapid cell degradation in the electrolyte or anode‐less Na batteries (a limited amount of electrolyte or Na‐ion reservoir is consumed in the first cycles).^[^ ^14^ ^]^ To compensate for the irreversible initial sodium loss, presodiation is an effective method to generate a stable SEI on the interlayer and mitigate the low initial CE during Na deposition–dissolution. Additionally, presodiation can passivate the interlayer by removing heteroatoms in the matrix structures, such as CNF, through oxidation and reduction reactions.^[^ ^12^, ^15^ ^]^ These heteroatoms can irreversibly react with active mobile ions in a battery, consuming a substantial amount and causing the initial formation and subsequent rupture/regeneration of unstable SEI.^[^ ^16^ ^]^

In this work, a 3D porous bimetallic CNF decorated with sodiophilic silver and sodiophobic copper (CuAg@C) and are used as an interlayer on a Cu foil current collector for Na deposition/dissolution. A less than 10 nm artificial SEI, consisting of organic components of RCONa and RCO_2_Na and inorganic components of Na_2_CO_3_ and Na_x_O_y_, is introduced onto CuAg@C by presodiation using *n*‐Butylsodium (*n*‐BuNa) (CuAg@C_Presod_). The morphology and chemistry of the interlayer and the artificial SEI were thoroughly analyzed by means of several analytical techniques such as scanning electron microscopy (SEM), X‐ray photoelectron spectroscopy (XPS), and Raman Spectroscopy. It was found that the presodiation process significantly improved the initial CE of the cells by pre‐reacting with defective sites in the CuAg@C and reducing irreversible side reactions during cycling. CuAg@C_Presod_ enabled a stable sodium plating/stripping process on the Cu current collector for over 6500 h with an average CE over 99.6%.

## 2 Results and Discussion

**Figure 1:**
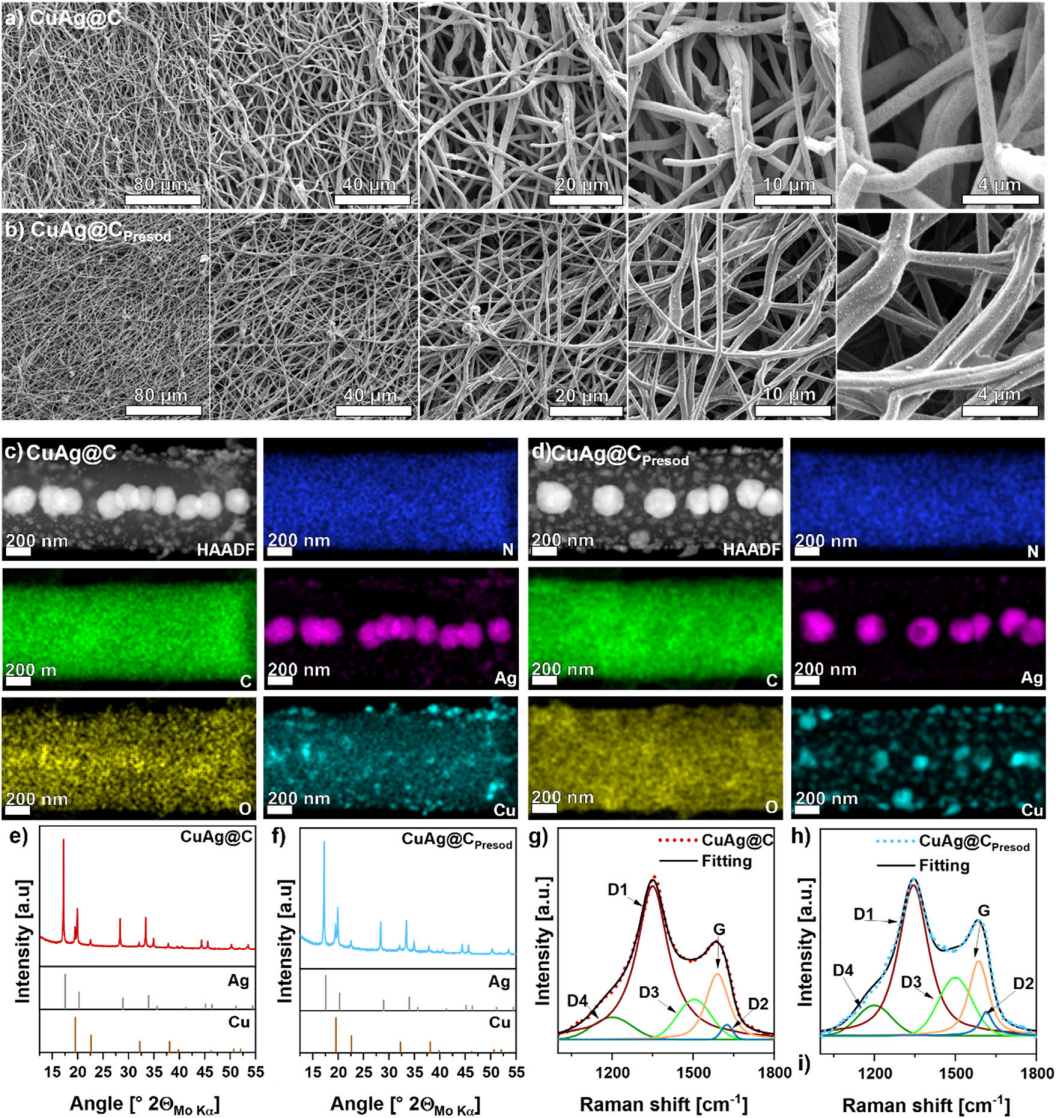
SEM images of a) CuAg@C and b) CuAg@C_Presod_ at different magnifications. STEM‐EDS maps of c) CuAg@C and d) CuAg@C_Presod_ showing the field of view and the element distribution for C, O, N, Ag, and Cu. Quantification results are provided in Table S1, Supporting Information. XRD patterns of e) CuAg@C and f) CuAg@C_Presod_ were measured with Mo–Kα radiation in transmission mode. Cu (Inorganic crystal structure database (ICSD) 136042) and Ag (ICSD 22434) are reference patterns. Raman spectra of g) CuAg@C and h) CuAg@C_Presod_ measured in an ECC‐Opto‐Std cell to avoid air contamination. Spectral parameters for the Raman fitting are listed in Table S2, Supporting Information.

The CuAg@C material was prepared as detailed in the experimental Section and previous work.^[^ ^12^ ^]^ Briefly, a fiber was first produced via electrospinning. The electrospun fiber was then stabilized at 250 °C in an oxygen atmosphere, followed by an Ar/H_2_ atmosphere reduction at 500 °C. Subsequently, the sample was presodiated in an *n‐*BuNa solution in THF/hexane to obtain the artificial SEI‐coated material, referred as CuAg@C_Presod_. To investigate the overall morphology of CuAg@C and CuAg@C_Presod_, SEM images at different magnifications were captured for both samples, as shown in **Figure** **1**a,b. A randomly distributed 3D fiber structure with large porosity is observed in both cases. This structure helps to alleviate stress during plating and stripping, thereby improving the cycling stability of the material.^[^ ^6^, ^7^ ^]^ Additionally, the presodiation does not significantly alter the fiber morphology, as both samples exhibit an average fiber diameter of 600 nm with a similar distribution trend.

The distribution of various elements within the fiber radius, especially Ag and Cu, was characterized by scanning transmission electron microscopy coupled with energy‐dispersive X‐ray spectroscopy (STEM‐EDS), as depicted in Figure 1c,d. The EDS mappings for C, O, and N show that these elements are homogeneously distributed throughout the entire fiber, commonly observed for CNF materials prepared via electrospinning of polyacrylonitrile.^[^ ^17^, ^18^ ^]^ Although Na EDS maps (Figure S1, Supporting Information) suggest that Na is present in both samples, a quantitative analysis (Table S1, Supporting Information) reveals the atomic fraction of Na in CuAg@C is only about 0.3 at%, which could be a result of surface contamination. In contrast, 10.1 at% Na is observed for CuAg@C_Presod_, suggesting the formation of Na‐rich phases on/in the presodiated interlayer. Furthermore, oxygen concentration increases from 3.4 at% for CuAg@C to 10.5 at% for CuAg@C_Presod_.

**Figure 2:**
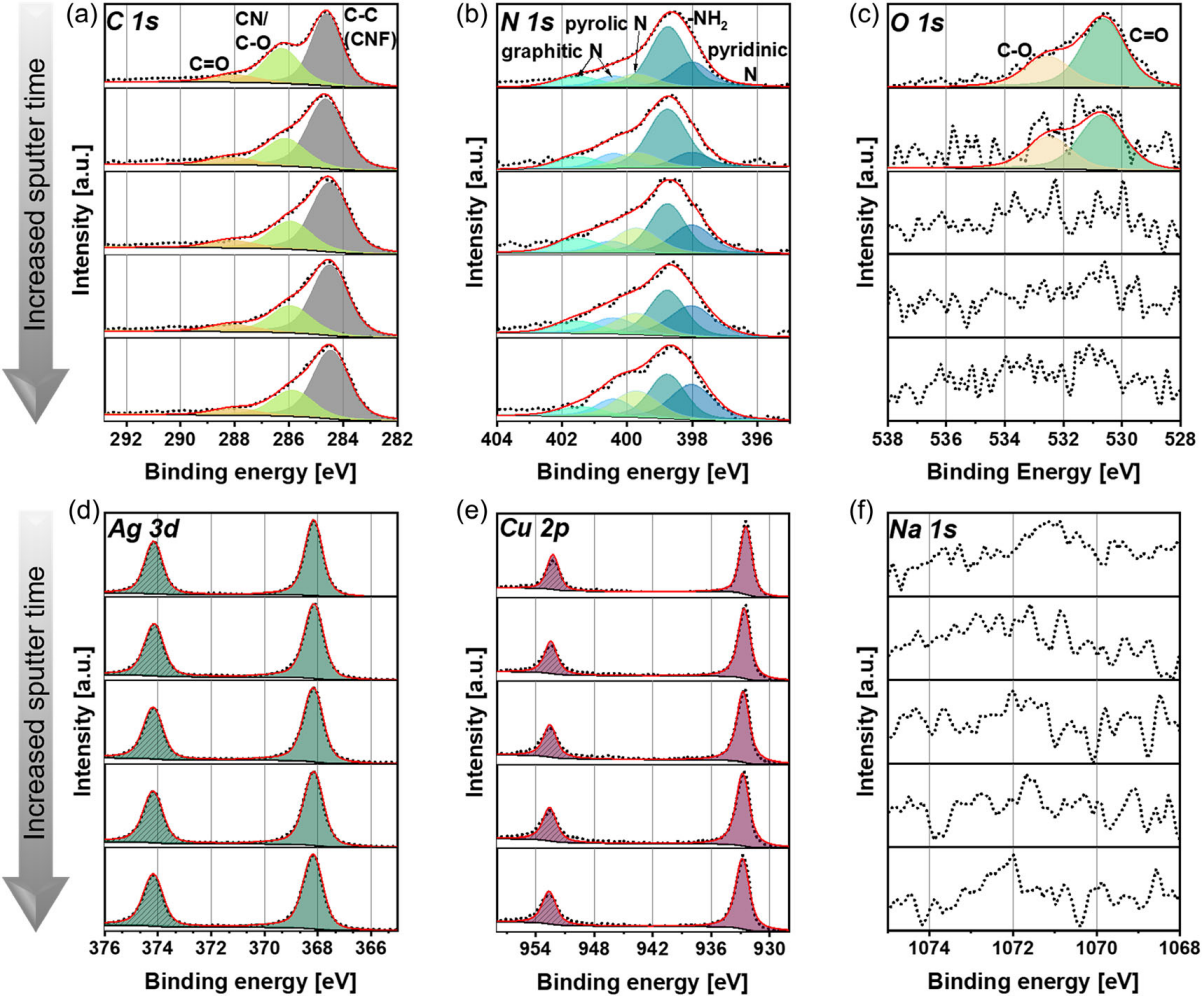
a) C 1*s*, b) N 1*s*, c) O 1*s*, d) Ag 3*d*, e) Cu 2*p,* and f) Na 1*s* XPS spectra of CuAg@C on the surface and after 10, 500, 1041, and 2002 s sputtering time. The spectra for CuAg@C are referenced for pyridinic N at 398.0 eV. Detailed measurement parameters can be found in Table S3, Supporting Information. The quantification results and fitting parameters can be found in Table S4, Supporting Information. The survey measurement can be found in Figure S4, Supporting Information. All spectra are normalized, with the highest signal in each spectrum set to 1.

The presence of oxygen in CuAg@C can be attributed to the synthesis process, during which an oxidation step at 250 °C is performed to stabilize the as‐spun interlayer. This step can introduce oxygen to the carbon matrix in the form of functional groups such as ketones, phenols, and lactones. Due to the low carbonization temperature of 500 °C, oxygen cannot be fully removed from the porous CNFs of CuAg@C.^[^ ^19^ ^]^ However, the XPS depth profiles in **Figure** **2** show that most of the oxygen was removed from the bulk of CNF, leaving 3.4 at% mainly on the surface of CuAg@C. In contrast, the increase in oxygen content for CuAg@C_Presod_ can be attributed to the decomposition of the solvent tetrahydrofuran (THF) in the *n*BuNa/hexane/THF solution during chemical sodiation, which contributes to the formation of the artificial SEI.^[^ ^20^ ^]^

The EDS mappings of Ag and Cu indicate that large silver particles are located at the center of the fiber, while smaller ones are on the surface. In contrast, small Cu particles are primarily located in the outer layers of the fiber rather than in its core. This alignment results from the differing behaviors of the two precursors in DMF. While Cu(OAc)_2_ remains dissolved, Ag^+^ from AgNO_3_ is reduced by DMF to form metallic Ag particles. Under the high electric field applied during the electrospinning process, these metallic Ag particles align in a chain‐like manner along the center of the fiber strands.^[^ ^12^ ^]^ The STEM‐EDS cross‐sectional maps further confirm the elemental distribution features described previously for C, O, N, Na, Ag, and Cu, as shown in Figure S2, Supporting Information. As a result, a sodiophilic–sodiophobic gradient is formed, extending from the core to the surface of the CNFs. The sodiophobic copper atoms in the outer fiber layers prevent sodium from accumulating as agglomerates on the fiber surface.^[^ ^10^ ^]^ Instead, sodium preferentially deposits in the sodiophilic silver regions.^[^ ^21^ ^]^ By integrating sodiophobic Cu in the outer fiber layers with sodiophilic Ag in the core, the top‐growth behavior of sodium on the interlayer is suppressed, thereby lowering the risk of dendrite formation during cycling.^[^ ^22^ ^]^

The Cu and Ag chemical states within the CuAg@C and CuAg@C_Presod_ were investigated using X‐ray diffraction (XRD), as shown in Figure 1e,f. In both cases, the XRD patterns represent a superposition of Cu and Ag reflections, with a noticeable overlay of about 20°.^[^ ^23^, ^24^ ^]^ No additional peaks are observed in the XRD pattern of CuAg@C_Presod_, implying that virtually no new crystalline components are formed within the fibers after presodiation. Furthermore, no reflections corresponding to ordered carbon are detected in the samples, indicating that the carbon in the CNFs exhibits a high degree of disorder.

To investigate the carbon structure of CuAg@C and CuAg@C_Presod_ Raman measurements were conducted, with the corresponding spectra shown in Figure 1g,h. Both spectra were fitted using the five‐band model described by Brubaker et al. enabling a more detailed analysis of the carbon structure.^[^ ^25^ ^]^ The spectral parameters for the Raman fitting can be found in Table S2, Supporting Information.

The G‐band corresponds to an ideal graphitic lattice (*E*_2g_ symmetry), while the D1 band is associated with A_1g_ symmetry and indicates disordered carbon. The D2 band is attributed to graphene layers on the surface, while the D3 and D4 bands relate to amorphous and heteroatom‐containing carbon, respectively.^[^ ^26^ ^]^ Comparing the area ratios of the D1 and G bands reveals that the *A*_D1_/*A*_G_ ratio decreases from 3.66 for CuAg@C to 3.09 for CuAg@C_Presod_ after presodiation. This reduction indicates a higher degree of graphitization for CuAg@C_Presod_. This finding is consistent with the fact that the electrical resistance, determined via DC polarization (Figure S3, Supporting Information), decreases from 333 MΩ for CuAg@C to 5 MΩ for CuAg@C_Presod_. The reduced electrical resistance of CuAg@C_Presod_ can be attributed to the additional heat treatment at 300 °C during the presodiation process. This step can remove heteroatoms from the bulk material in the form of H_2_O, CO, and CO_2_, thereby creating a more ordered carbon structure.^[^ ^19^ ^]^ However, these molecules on the surface of the fiber react with the presodiation agent *n‐*BuNa, contributing to the formation of the artificial SEI. This is also reflected in the (*A*_D3_ + *A*_D4_)/*A*_total_ ratio shown in Table S2, Supporting Information, indicating surface‐related disorder. A comparison of these ratios between CuAg@C and CuAg@C_Presod_, 0.21 versus 0.28, indicates that the presodiated sample CuAg@C_Presod_ exhibits a greater degree of surface‐related disorder, suggesting a change in the surface layer.

Since the Raman measurements did not provide further insights into the surface chemistry, XPS and XPS depth profile measurements were conducted on the uncycled CuAg@C and CuAg@C_Presod_ samples to gain a deeper understanding of the surface composition. Figure 2 shows the XPS spectra of the surface for the elements C 1*s*, N 1*s*, O 1*s*, Ag 3*d*, Cu 2*p*, and Na 1*s* of CuAg@C, as well as the depth profiles after 10, 500, 1041, and 2002 s of sputtering time. The C 1*s* spectrum of CuAg@C (Figure 2a) presents two distinct signals at 284.61 and 286.3 eV, respectively, corresponding to the C—C environment and the strongly overlapping contribution of C=N, C—N, and C—O. In addition, the small signal at 288.0 eV can be attributed to carbon in a C=O environment. The oxygen of the C—O and C=O components are attributed to oxygen in CuAg@C that was not completely removed during carbonization. Figure 2a shows that no new signals can be detected with increased sputtering time. However, after 10 s, the signal at 286.3 eV (C=N, C—N, and C—O) to 286.1 eV. Since C=N and C—O characteristically appear at 286.5 eV, and C—N at 286 eV, this indicates a change in the relative ratio of the three contributions, favoring the C—N component.^[^ ^27^, ^28^ ^]^ Moreover, this shift becomes more pronounced with increasing sputtering time. After 500 s of sputtering, the signal shifts to 285.9 eV and remains there regardless of any further increase in the sputtering time. This implies that the fiber surface exhibits a lower degree of aromatization than the fiber core due to the reduction step in Ar/H_2_ flow, which can be further confirmed by the N 1*s* spectrum shown in Figure 2b.

The N 1*s* spectrum was fitted with five distinct components. At 398 eV, nitrogen in a pyridinic configuration belonging to the N‐doped CNFs is observed. The signal at 398.8 eV can be attributed to ‐NH_2_ groups, suggesting that the sample is not fully cross‐linked.^[^ ^29^ ^]^ The origin of this component is the hydrogenation of a C≡N group present in polyacrylonitrile during the reduction of the composite material in Ar/H_2_.^[^ ^18^ ^]^ A pyrrolic nitrogen component is also detected at 399.7 eV, along with two signals assigned to graphitic nitrogen at 400.4 and 401.5 eV. The graphitic nitrogen is located at different sites within the polymer, leading to variations in the binding energies, as described by Lazar et al.^[^ ^30^ ^]^ After 10 s of sputtering, a decrease in the signal attributed to ‐NH_2_ and a reduction in its quantitative contribution to the surface composition are observed (Table S4, Supporting Information). This trend continues with increasing sputtering time, and starting from 1041 s, more nitrogen associated with pyridinic N is detected than with ‐NH_2_, confirming the increased degree of aromatization from the surface to the bulk of CNFs.

The O 1*s* spectrum is shown in Figure 2c, with two distinct components C—O at 532.5 eV and C=O at 530.6 eV identified on the surface. These oxygen‐containing components in CuAg@C originate from the oxidation step during synthesis, as described in Figure 1. However, upon sputtering CuAg@C, it becomes evident that the oxygen components are only surface bonded. As shown in Table S4, Supporting Information, less than 2% oxygen is detected after just 10 s of sputtering, and no oxygen is visible in the sample after 500 s. A distinct trend is observed for Ag and Cu, as displayed in Figure 2d,e. Both elements are characterized by a doublet, caused by the spin‐orbit coupling of *p*‐ and *d*‐ orbitals, in contrast to thse *s*‐orbital. The two peaks have the same full width at half maximum and appear in a specific ratio, depending on the orbital type. The more intense signal for Ag (Ag 3*d*_5/2_) is at a binding energy of 386.2 eV and for Cu (Cu 2*p*_3/2_) at 932.4 eV. Silver is present on the surface in higher concentrations compared to copper, 9.9 versus 5.6 at%, respectively. However, the Ag concentration steadily declines during sputtering from 7.1 at% at 500 s to 4.6 at% at 2002 s. From the sputtering time of 500 s, Cu became the dominant element, with a higher proportion of 9.6 at% than silver, a trend that persisted until 2002 s of sputtering time, when copper reached 6.9 at%. Considering the STEM‐EDS data, the sputtering depth has not yet reached the silver particles located in the center of the fiber, while copper is more present in the outer layers. This indicates the presence of a sodiophobic–sodiophilic gradient from the surface to the core of the CuAg@C fiber. Expectedly, no Na 1*s* signal is detected in the pristine non‐sodiated sample, as shown in Figure 2f.

**Figure 3:**
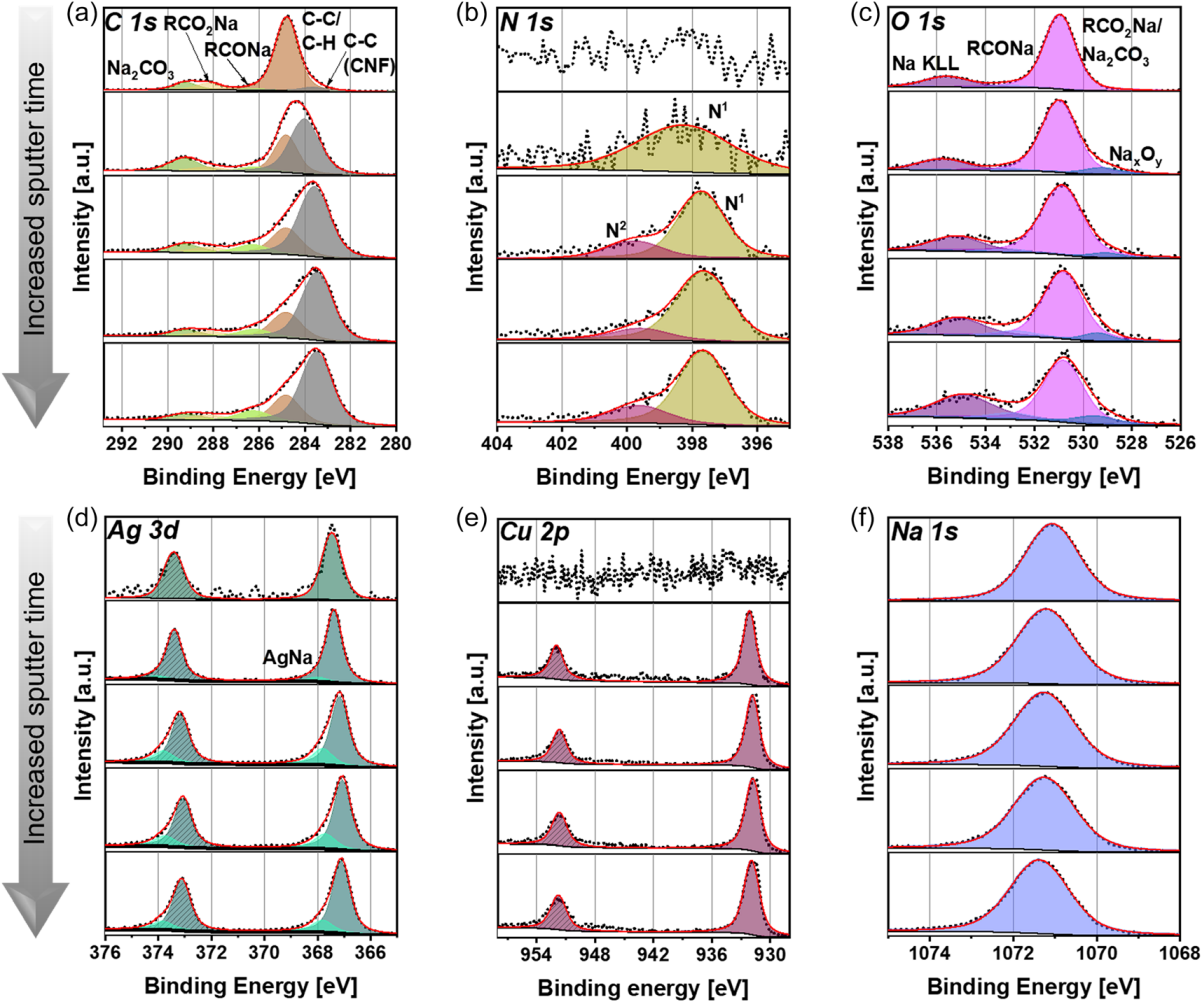
a) C 1*s*, b) N 1*s*, c) O 1*s*, d) Ag 3*d*, e) Cu 2*p*, and f) Na 1*s* XPS spectra of CuAg@C_Presod_ on the surface and after 10, 500, 1041, and 2002 s sputter time. The spectra for CuAg@C_Presod_ are referenced for C*—*C/C*—*H to 284.8 eV. Detailed measurement parameters can be found in Table S3, Supporting Information. The quantification results and fitting parameters can be found in Table S5, Supporting Information. The survey measurement can be found in Figure S5, Supporting Information. All spectra are normalized, with the highest signal in each spectrum set to 1.

The same surface analyzes as those conducted for CuAg@C were performed for CuAg@C_Presod_ to investigate the evolution of the sample induced by the presodiation. The decomposition of *n‐*BuNa and the reaction of *n‐*BuNa with the carbon chains of the fibers, as well as the solvents hexane and THF, resulted in the detection of four new C 1*s* signals on the surface of CuAg@C_Presod_, as presented in **Figure** **3**a. The largest signal is observed at 248.8 eV, attributed to carbon in a C—C/C—H environment.^[^ ^31^ ^]^ A small amount of carbon in the RCONa component is present, indicated by a signal at 286.2 eV. The other two signals are assigned to RCO_2_Na and Na_2_CO_3_ at 288.2 and 289.2 eV, respectively, indicating that the artificially produced SEI consists of organic and inorganic components.

In addition to the new peaks, a peak at the binding energy of 283.6 eV, corresponding to the C—C environment of CNFs, is also observed as for CuAg@C, but with smaller intensity. The small signal shift of this component compared to the untreated material can be attributed to the presence of the artificial SEI. Between the artificial SEI and the bulk material, a potential gradient is formed, causing the components associated with the artificial SEI to shift to higher binding energies. As a result, referencing components from the artificial SEI, such as C—C/C—H, for component assignment can create the appearance that the bulk material has shifted to lower binding energies.^[^ ^32^ ^]^ Although no new components are detected in the *C 1s* depth profile with increased sputtering time, a significant change in the component ratios is observed. As shown in Table S5, Supporting Information, the concentrations of components associated with the artificial SEI, such as Na_2_CO_3_ and RCO_2_Na, decrease from 4.4 to 3.1 at% and from 4.01 to 2.40 at%, respectively, which falls within the error margin of the XPS quantification. Similarly, the C—C/C—H component decreases from 42.7 to 12.9 at% over the entire sputtering time. In contrast, the intensity of the carbon component attributed to bulk carbon increases significantly, from 2.5 at% at the surface to 40.2 at% after 2002 s sputtering time. This increase suggests a thin SEI layer, as signals from the bulk material become detectable after only a short sputtering time. This observation is further supported by the N 1s spectrum shown in Figure 3b. Nitrogen is mostly located within the bulk of the material and is not detected on the surface of CuAg@C_Presod_. Nevertheless, a nitrogen signal emerges after 10 s of sputtering, with its intensity increasing progressively with further sputtering. However, due to the aforementioned shifts, it is not possible to distinguish the nitrogen signal precisely, as it may include sodiated species and those present in CuAg@C.

In the oxygen spectra of CuAg@C_Presod_ shown in Figure 3c, much higher intensities of the signals are seen compared to CuAg@C, with over 26 at% surface concentration. However, these are not the same components as those observed for CuAg@C. As described in Figure 1, additional oxygen‐containing components are released from the material due to the extra heating step during presodiation. These components can then react with *n‐*BuNa and deposit on the surface. Furthermore, additional oxygen is introduced into the system through the decomposition of THF. Consistent with the results from the C 1*s* spectrum in Figure 3a, the stronger signal at 531.0 eV can be linked to oxygen in a C=O environment in RCO_2_Na and Na_2_CO_3_, while the less intense signal at 533.0 eV is assigned to oxygen in a C—O environment. Another signal at 535.6 eV originating from a Na KLL Auger electron is also identified. Upon examination of the depth profiles, an additional signal attributed to oxygen in a Na_x_O_y_ environment with a contribution of 1–2 at% appears in addition to these observed signals (Table S5, Supporting Information). During the heating step of the presodiation, oxygen can be released from the fiber in the form of H_2_O, where it reacts with Na to form NaOH. At elevated temperatures, NaOH can further react with additional sodium to form Na_x_O_y_.^[^ ^33^ ^]^ An alternative explanation is that the observed feature could be sputter‐induced damage. However, since the concentration of the component does not increase further after 10 s of sputtering, this option is considered unlikely in this case.

In conjunction with the Ag 3*d* and Cu 2*p* spectra shown in Figure 3d,e, information regarding the thickness of the artificial SEI layer can be estimated. While no signals for nitrogen (N 1*s*) or copper (Cu 2*p*) are detected at the surface, a signal for silver (Ag 3*d*) is visible at 367.5 eV. The absence of copper and nitrogen signals on the surface, along with the detection of silver, suggests that the artificial SEI has an approximate thickness of less than 10 nm, corresponding to the information depth of the XPS instrument used. Furthermore, this observation is confirmed by the depth profiles, as copper and nitrogen become detectable after a sputtering time of 10 s. These findings are consistent with the results in Figure 1, where no significant expansion of the fibers was observed after presodiation.

**Figure 4:**
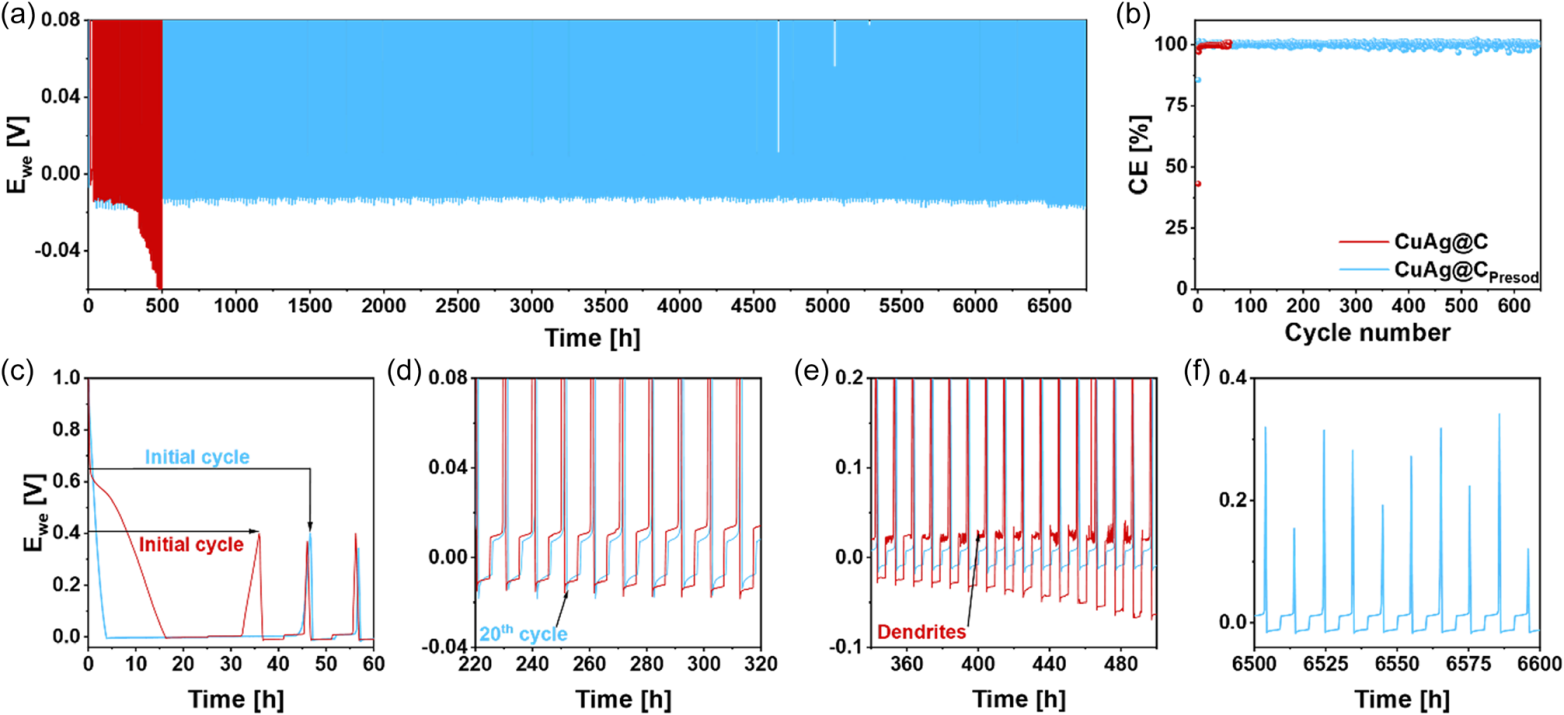
a) Voltage versus time profiles of CuAg@C and CuAg@C_Presod_ during Na plating/stripping tests. b) Relevant Coulombic efficiencies for the voltage versus time profile depicted in (a). zoomed‐in sections from (a) at different periods of c) initial 60 h, d) example of stable cycling, e) dendrite formed for CuAg@C, and f) stable cycle of CuAg@C_Presod_ over 6600 h.

As previously discussed, the shift observed in the Ag 3*d* spectrum at 367.5 eV, compared to metallic silver at 386.2 eV, can be attributed to a potential gradient between the SEI and the bulk.^[^ ^34^ ^]^ However, with increasing sputtering time, fitting the Ag 3*d* spectrum with only one species is not possible, requiring the introduction of a second species to fit the experimental data. This second component is shifted to slightly higher binding energies compared to the signal attributed to silver. This additional signal suggests the formation of an AgNa alloy, as it would be similarly shifted to higher binding energies as an AgLi alloy.^[^ ^35^ ^]^ The Na 1*s* spectrum of CuAg@C_Presod_, shown in Figure 3f, further confirms the presence of sodium inside the material, and not only on the surface. Due to the overlap of various components in the Na 1*s* spectrum, a detailed peak analysis is not feasible. Instead, the measurement determined sodium content within the error margin of XPS with 11.8 at% at the surface and 10.3 at% after 2002 s of sputtering. To evaluate the impact of the artificial SEI on electrochemical performance, cycling tests were performed to compare the stability of CuAg@C and CuAg@C_Presod_ during sodium plating and stripping. Metallic Na is used as the reference and counter electrode, while CuAg@C or CuAg@C_Presod_ functions as the working electrode, being placed on a planar Cu foil current collector. As shown in **Figure** **4**a,c–f, both materials were initially plated at a current density of 0.1 mA cm^−2^ for 25 h, followed by stripping at 0.1 mA cm^−2^ until the voltage reached 0.4 V. After the formation cycle, the current was increased to 0.5 mA cm^−2^ for both plating and stripping, while the plating time was reduced to 5 h. The high capacity of 2.5 mA cm^−2^ per cycle ensures a fast evaluation of the cycling stability due to the increased possibility of Na dendrite formation, enabling rapid observation of distinctions between the samples.

Significant differences between the two samples are observed during the formation cycle, magnified in Figure 4c. The potential of the CuAg@C cell drops quickly to 0.7 V, after which it gradually declines to negative voltages after 16.5 h, suggesting additional reactions in the plating mechanism. These reactions could involve reactions of Na with heteroatoms like O and H in CuAg@C, as previously indicated by Raman and XPS analyzes, and electrolyte decomposition reactions forming the SEI.^[^ ^36^, ^37^ ^]^ When the cell potential reaches negative values, sodium precipitates on the CuAg@C interlayer. The initial stripping process of CuAg@C lasts only about ten h, compared with the long plating process, resulting in an initial CE of 43% (cf. Figure 4b).

In contrast to CuAg@C, the potential of the CuAg@C_Presod_ cell reaches a negative value within 4 h of plating during the initial cycle, suggesting that less Na^+^ is consumed in irreversible reactions due to the presence of an artificial SEI. This improvement is reflected in a notably higher initial CE of 86%. Further cycling tests, shown in Figure 4d,e, reveal that the CE of CuAg@C increases rapidly over the first few cycles, surpassing 99% by the fifth cycle and eventually approaching CEs over 99.3% for the rest of the cycles (Figure 4b), which indicates a high degree of reversibility in the sodium plating/stripping process. However, after ≈360 h, a notable rise in overpotential is observed, accompanied by the onset of dendrite formation, ultimately leading to cell failure at around 500 h. Conversely, CuAg@C_Presod_ demonstrates CEs over 99.5 % from the second cycle, signifying highly reversible sodium plating/stripping.

Unlike CuAg@C, CuAg@C_Presod_ shows no increase in overpotential or signs of dendrite formation, even after more than 6500 h of cycling, as displayed in Figure 4f. These findings demonstrate the long‐term stability of CuAg@C_Presod_ and are further confirmed by impedance measurements of CuAg@C and CuAg@C_Presod_ cells as the Nyquist plots shown in Figure S6h, Supporting Information. While CuAg@C initially shows a relatively low overall resistance, it increases rapidly upon cycling, doubling after 30 cycles. This increase in resistance indicates an unstable surface, as supported by the XPS results shown in Figure 6, which suggest that the surface continuously thickens due to further decomposition reactions. The resistance continues to rise and doubles again after 40 cycles. This rapid increase in the cell's overall resistance highlights material failure. In contrast, the overall resistance of CuAg@C_Presod_ is more constant during cycling, and no significant rise in resistance is observed during the first 200 cycles. Although the resistance increases gradually thereafter, the total increase remains below 100 % over 600 cycles, highlighting the good stability of CuAg@C_Presod_.

The rate performance of CuAg@C and CuAg@C_Presod_ was examined at the current densities from 0.1 to 2.5 mA cm^−2^ for CuAg@C and 3.5 mA cm^−2^ for CuAg@C_Presod_, respectively, as the results are presented in Figure S7, Supporting Information. To ensure that both samples were evaluated solely for their sodium plating/stripping stability, two initial formation cycles were carried out at 0.1 mA cm^−2^ for 45 h, followed by stripping to 0.6 V. In the subsequent cycles, the capacity was fixed at 2.5 mAh cm^−2^, and the current density was increased incrementally every five cycles, starting at 0.1 mA cm^−2^. CuAg@C maintained stable cycling up to a current density of 2.0 mA cm^−2^. The CuAg@C_Presod_ sample exhibits better rate performance and stable cycling up to 3.0 mA cm^−2^, while failure occurs at a current density of 3.5 mA cm^−2^. The improved performance in CuAg@C_Presod_ highlights the benefits of presodiation, leading to higher initial CE, enhanced cycling stability, and rate performance compared to CuAg@C. To evaluate the stability of CuAg@C_Presod_ under higher current densities, an additional test was conducted against sodium at increased current rates, as shown in Figure S8, Supporting Information. Therefore, the current density during the formation cycle was raised to 0.25 mA cm^−2^ and subsequently increased to 1.25 mA cm^−2^ in the following cycles. The deposition time was adjusted such that a total areal capacity of 2.5 mAh cm^−2^ of sodium was deposited in each cycle. Even at this elevated current density, the material demonstrated excellent cycling stability for over 140 cycles with Coulombic efficiencies exceeding 99.5%.

**Figure 5:**
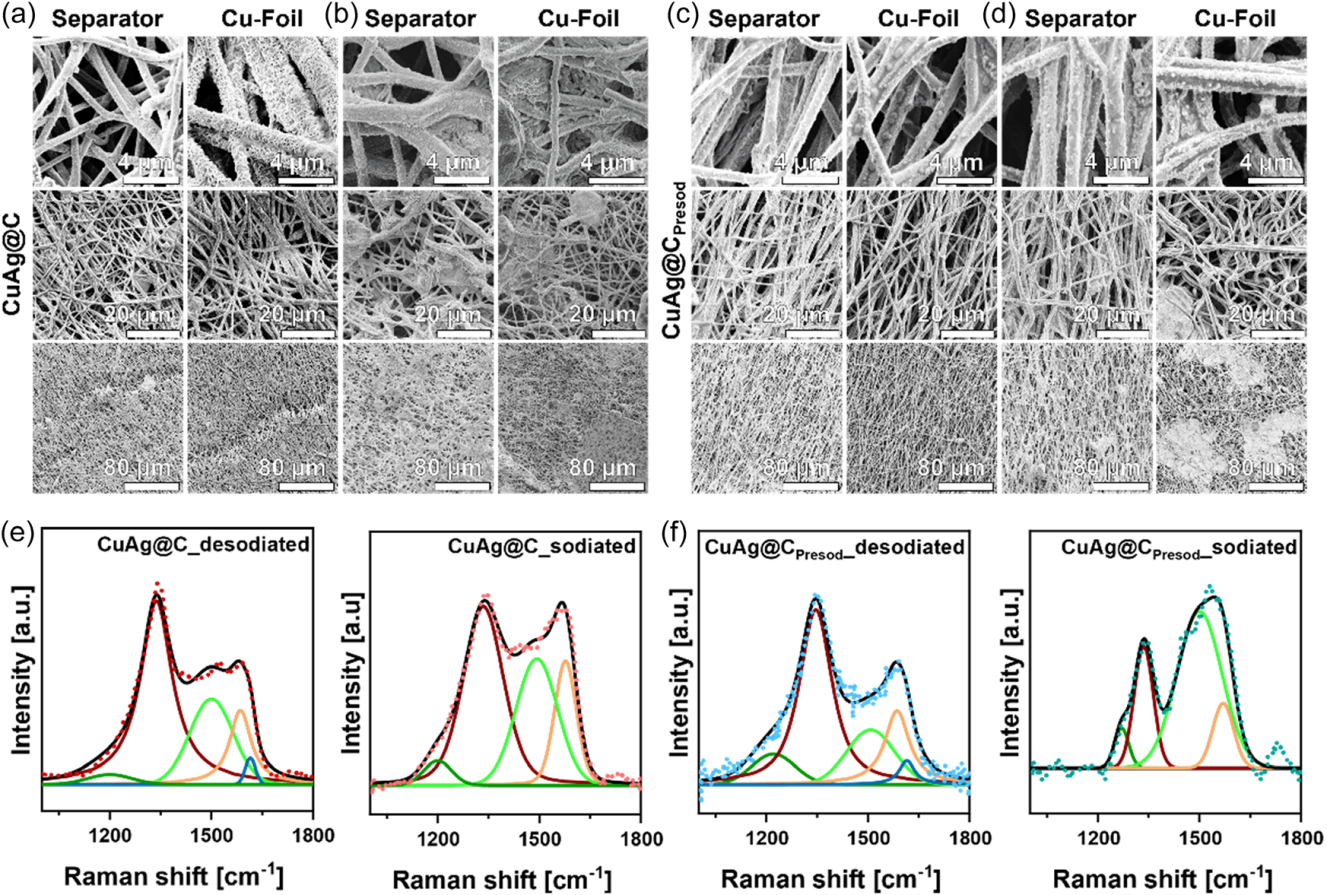
SEM images of a,b) CuAg@C and c,d) CuAg@C_Presod_ depicting the sodiated (left) and desodiated state (right) for the side facing the separator (left) and the side facing the Cu foil (right). The samples underwent a formation cycle at 0.1 mA cm^−2^ for 25 h and subsequently cycled five times at a current density of 0.5 mA cm^−2^ at room temperature. e,f) Raman spectra of the two samples measured in an ECC‐Opto‐Std cell for air protection, respectively. Corresponding fitting parameters are listed in Table S6 and S7, Supporting Information. The CuAg@C and CuAg@C_Presod_ underwent a formation cycle at 0.1 mA cm^−2^ for 25 h, and were further cycled five times at a current of 0.5 mA cm^−2^.

SEM images were taken after cycling to better understand the difference in cycling behavior of CuAg@C and CuAg@C_Presod_. These images were obtained in both sodiated and desodiated states to provide insights into the evolution of the structure of the materials during Na plating/stripping. Additionally, both sides of the interlayer‐facing the separator and facing the copper foil were examined. This approach enabled a comprehensive analysis of sodium deposition and accumulation on both sides, which can significantly impact the cycling stability and CE of the materials. As illustrated in **Figure** **5**a, the desodiated state of CuAg@C exhibits no discernible Na agglomerations after five cycles. In contrast, in the sodiated state presented in Figure 5b, multiple Na agglomerations are evident on both sides, oriented toward the copper current collector and the side oriented toward the separator. Such an uneven deposition process could result in sodium dendrites and thereby increase the risk of short circuit by penetrating the separator. The deposition of sodium on both sides of the CuAg@C interlayer indicates that the function of the fiber as a protective barrier is impaired, facilitating sodium accumulation in regions and increasing the probability for dendrite growth and cell failure.^[^ ^6^, ^7^ ^]^

The SEM images of CuAg@C_Presod_ in the desodiated state (Figure 5c) did not reveal any discernible metallic Na agglomerations, in contrast to the observations made for CuAg@C. This observation is consistent with the high CE observed for CuAg@C_Presod_, demonstrating that nearly all plated sodium is effectively stripped in each cycle. In the sodiated state, CuAg@C_Presod_ displays a more controlled and localized sodium plating process than CuAg@C, as illustrated in Figure 5d. The accumulation of sodium is observed to occur exclusively on the side of the material that is in contact with the copper foil, while the other side in contact with the separator remains essentially free of metallic Na. In addition to the process by which sodium is preferentially directed toward the sodiophilic silver in the center of CNFs, the observed sodium accumulations on CuAg@C_Presod_ have the potential to grow into the open pores between the carbon fibers, thereby maintaining electrical contact with the electrode. This allows the plated sodium to be fully utilized during subsequent stripping cycles. Moreover, the well‐preserved morphology of CuAg@C_Presod_ indicates that the material can tolerate the volume changes associated with the plating and stripping of sodium, thereby enhancing its stability for the sodium deposition/dissolution process. By confining the Na deposition on the Cu foil side, CuAg@C_Presod_ effectively reduces the risk of dendrite formation and preserves the function of the 3D structure as a protective barrier, preventing sodium penetration through the separator and enhancing overall Na deposition/dissolution stability.

Figure 5e,f illustrate the Raman spectra of CuAg@C and CuAg@C_Presod_ in both desodiated and sodiated states. The spectra were fitted according to the five‐band model proposed by Brubaker et al.^[^ ^25^ ^]^ The comparison of the previously analyzed ratios of *A*_D1_/*A*_G_ and (*A*_D3_ + A_D4_)/*A*_total_ of the uncycled materials with the ratios after cycling provides insights into the stability of the bulk material and the surface‐related disorder, respectively. In the sodiated state of CuAg@C, the *A*_D1_/*A*_G_ ratio decreases to 2.68 (Table S6, Supporting Information), reducing the degree of graphitization within the bulk material. In the desodiated state of the cycled CuAg@C, the *A*_D1_/*A*_G_ ratio returns to 3.64, comparable to that of the uncycled material, suggesting that the structure of the fiber remains stable. Conversely, the surface‐related disorder in the sodiated state increases significantly to 0.36, as indicated by the rising (*A*_D3_ + *A*_D4_)/*A*_total_ ratio. This increase can be attributed to SEI formation and sodium deposition. However, in the desodiated state, the surface disorder remains higher at 0.30 compared to the uncycled CuAg@C, suggesting that the process is not entirely reversible and that an SEI has formed. This observation is consistent with the impedance measurements from Figure S6, Supporting Information, which also demonstrate unstable cycling behavior for the CuAg@C sample, as the resistance increases with the higher cycle number.

In comparison, CuAg@C_Presod_ in the sodiated state exhibits the most substantial decrease in the *A*_D1_/*A*_G_ ratio, reaching 1.74 (Table S7, Supporting Information). Additionally, it displays the highest surface disorder, with a value of 0.67. These findings indicate that sodium interacts more homogenously with CuAg@C_Presod_, thereby preventing agglomeration at specific points. In contrast to CuAg@C, CuAg@C_Presod_ exhibits values of 2.9 for the *A*_D1_/*A*_G_ ratio and 0.28 for the surface‐related disorder after desodiation, with both ratios approaching the values observed in the uncycled CuAg@C_Presod_, which is in agreement with the results from the impedance measurements in Figure S6, Supporting Information.

**Figure 6:**
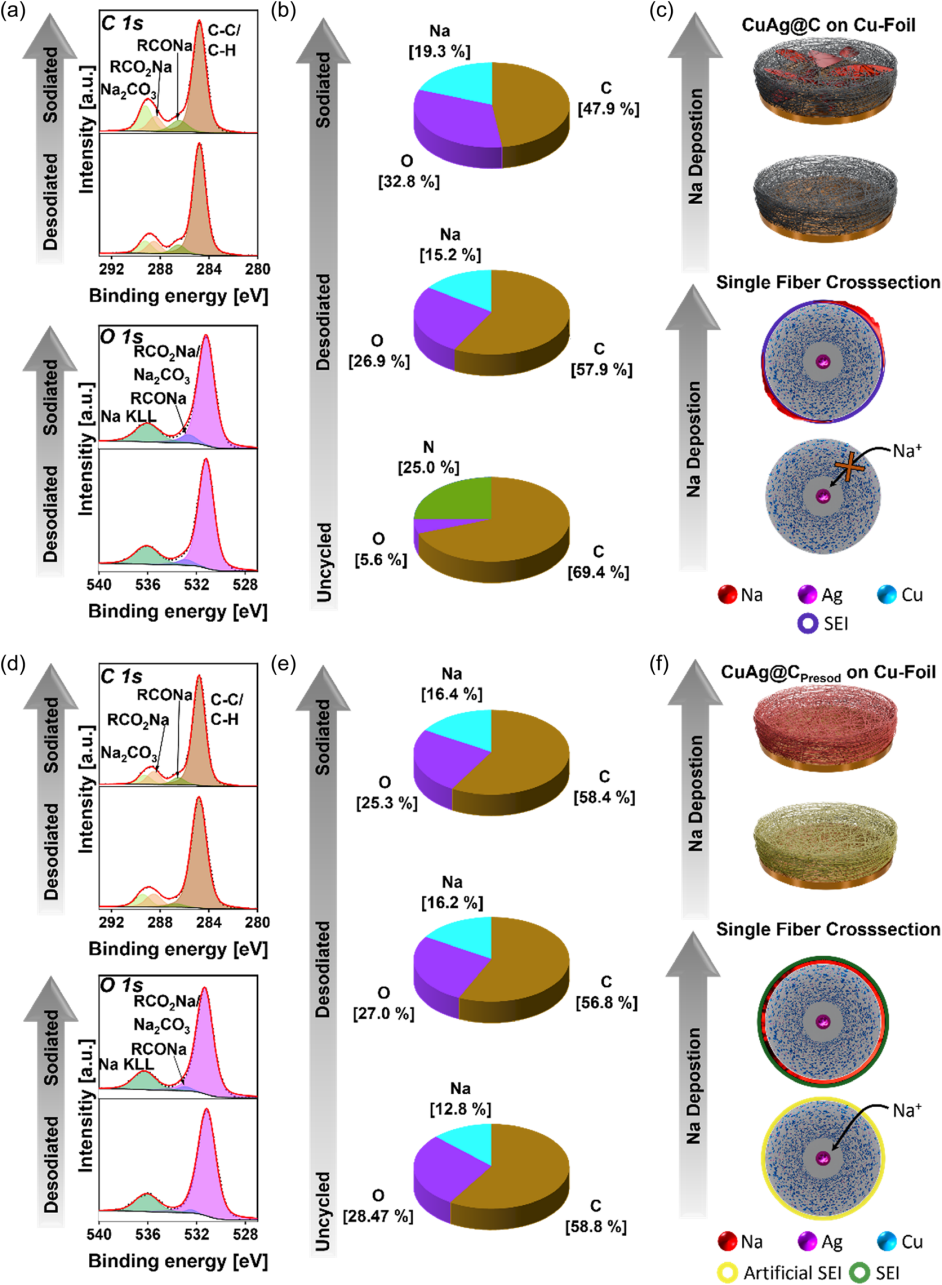
C 1*s*, O 1*s* XPS spectra, distribution of the elements C, O, and Na, and schematic illustration of the Na deposition behavior of a–c) CuAg@C and d–f) CuAg@C_Presod_, respectively. XPS spectra were obtained in the sodiated and desodiated states after the cells were initially cycled at 0.1 mA cm^−2^ for 25 h, and another five cycles at a current of 0.5 mA cm^−2^ at room temperature. Detailed measurement and fitting parameters can be found in Table S3, Supporting Information. F 1*s* and P 2*p* XPS spectra are presented in Figure S9, Supporting Information, and N 1*s*, Na 1*s*, Cu 2*p*, and Ag 3*d* XPS spectra are depicted in Figure S10, Supporting Information. Survey measurements for all four samples can be found in Figure S11, Supporting Information. All spectra are normalized, with the highest signal in each spectrum set to 1. Quantification results and fitting parameters of the XPS spectra are listed in Table S8, Supporting Information.

To analyze the SEI formed during cycling, XPS measurements were conducted on CuAg@C and CuAg@C_Presod_ in both their sodiated and desodiated states after cycling. The C 1*s*, O 1*s* XPS spectra of CuAg@C in its desodiated and sodiated states are shown in **Figure** **6**a. Four distinct signals are observed in the C 1*s* spectrum in both states. The most prominent peak at 284.8 eV corresponds to carbon in a C—C/C—H environment, a common feature of SEIs formed in organic electrolytes. The second peak at 286.5 eV is attributed to carbon in RCONa, while the peaks at 288.5 and 289.2 eV correspond to carbon in RCO_2_Na and Na_2_CO_3_ environments, respectively. These species are also reflected in the O 1*s* spectrum, where the signal at 532.9 eV indicates oxygen in an RCONa bond, and the signal at 531.2 eV corresponds to RCO_2_Na and Na_2_CO_3_. In the F 1*s* and P 2*p* spectra in Figure S9, Supporting Information of CuAg@C_desodiated and CuAg@C_sodiated, a dominant signal suggests the presence of electrolyte residues on the surface of both samples. Although the SEI of CuAg@C in both the sodiated and desodiated states consists of the same components, their relative content varies noticeably. For instance, as shown in Table S8, Supporting Information, a change in the contribution of the Na_2_CO_3_ component from 4.6 at% in the desodiated state to 7.0 at% in the sodiated state and the C—C/C—H component from 39.7 to 30.2 at% is observed. These variations indicate an unstable SEI that undergoes ongoing compositional changes throughout cycling, likely induced by the dissolution of SEI components and the different cut‐off voltages used during the plating and stripping processes.^[^ ^38^ ^]^

The instability of the SEI in CuAg@C is further evident in the elemental composition of the surface in both states, as depicted in Figure 6b. On the sodiated CuAg@C surface, the SEI exhibits a decrease in carbon content accompanied by an increase in oxygen and sodium levels compared to the desodiated CuAg@C surface. This shift in composition indicates SEI instability, suggesting continuous reformation of the interphase during cycling. Additionally, the absence of signals in the Ag 3*d*, Cu 2*p*, and N 1*s* spectra, as shown in Figure S9, Supporting Information, suggests that the SEI formed on the surface of CuAg@C is thicker than 10 nm, exceeding the detection depth of the instrument.

Figure 6c schematically illustrates the proposed sodium deposition mechanism on CuAg@C. In principle, sodium ions are expected to migrate toward the sodiophilic silver core located at the center of the fiber. This migration is anticipated to reduce top‐growth behavior, which is crucial for minimizing the formation of dendrites. The silver core is thought to be pivotal in guiding the sodium ions, directing them away from the fiber surface and toward the core, thereby promoting uniform deposition. However, as demonstrated previously, the presence of numerous defects within the structure significantly interferes with this ideal behavior. These defects act as localized trapping sites for sodium ions, which are quickly intercepted and nucleated at these sites. This leads to the rapid aggregation of sodium on the surface of the fiber, hindering the migration of ions toward the silver core. Consequently, sodium agglomerates form across the interlayer instead of being concentrated in the central core. This uniform distribution of sodium increases the likelihood of dendrite formation, which can compromise the integrity of the cell, ultimately leading to cell failure.

Figure 6d shows the C 1*s* and O 1*s* XPS spectra of CuAg@C_Presod_ in the sodiated and desodiated states after the initial cycles. In both states, components characteristic of the artificial SEI, previously identified on uncycled CuAg@C_Presod_, are observed. These include RCONa at 286.5 eV, RCO_2_Na at 288.5 eV, and Na_2_CO_3_ at 289.3 eV in the C 1*s* spectra. The corresponding signals in the O 1*s* spectra are observed at 531.2 eV, attributed to C—O groups, and at 532.9 eV, attributed to C=O groups. The Na 1*s* spectrum shown in Figure S10, Supporting Information confirms the presence of sodium in the SEI. Together with the quantification results from Table S8, Supporting Information, it is evident that the surface of the cycled samples contains 13.9 at% sodium, compared to 11.8 at% in the artificial SEI. This increase can be attributed to electrolyte residues on the surface, as illustrated in Figure S9, Supporting Information. The elemental composition of C, O, and Na presented in Figure 6e reveals that the SEI on CuAg@C_Presod_ undergoes only minimal changes during cycling, as measured within the information depth of less than 10 nm. The elemental distributions in the sodiated and desodiated states deviate only slightly from those of the initial artificial SEI, indicating its excellent stability throughout cycling. The initially thin artificial SEI is composed of organic components (RCO_2_Na, RCONa) and inorganic components (Na_x_O_y_, Na_2_CO_3_), resulting from the chemical sodiation reaction between *n‐*BuNa/hexane/THF solution and the heteroatoms (e.g., H and O) in the bimetallic CNF interlayer. These compounds can significantly enhance the overall conductivity of CuAg@C_Presod_ while maintaining the structural integrity of artificial SEI due to organic components. As illustrated in Figure 6f, the artificial SEI plays a crucial role in promoting the attraction of sodium ions Na to the sodiophilic silver core within the CNFs. This interaction effectively mitigates the formation of sodium agglomerates on the fiber surface by facilitating a more uniform ion distribution. The artificial SEI aids in directing sodium ions toward the silver core, thereby improving the control over the deposition process. In addition, the artificial SEI enhances the functionality of the distinctive structural features of the 3D bimetallic CNF interlayer, including the sodiophilic–sodiophobic gradient, high porosity, and large surface area with anisotropically distributed CNFs, all of which are advantageous for long‐term cycling applications. As shown in Table S9, Supporting Information, this results in improved cyclability compared to other modifications reported in the literature for stabilizing the Na deposition/dissolution process.^[^ ^12^, ^39^, ^40^ ^]^

## 3 Conclusion

This study evaluates the Na plating/stripping stability of a 3D porous carbon interlayer with (CuAg@C_Presod_) or without (CuAg@C) artificial SEI, especially focusing on the surface chemistry of the samples in different cycling states. Ideally, the Na plating/stripping stability is enhanced by the synergistic effects of the sodiophilic‐sodiophobic Ag–Cu gradient in individual fibers and the high porosity and surface tension of the CNF matrix. However, due to the low carbonization temperature of the interlayer, CuAg@C exhibits a highly disordered carbon structure with heteroatoms of O and H. As a result, the unique structural features of CuAg@C are not fully utilized for long‐term Na deposition and dissolution, and the sample has a low initial CE of 43% and fast cell failure at a cycling time of about 500 h due to irreversible side reactions and Na “top‐growth” on the interlayer. In contrast, presodiated CuAg@C using an *n‐*BuNa/hexane/THF solution can reduce the content of heteroatoms by redox reactions, resulting in increased overall conductivity and an artificial SEI thinner than 10 nm consisting of RCO_2_Na, ROCNa, Na_2_CO_3_, and Na_x_O_y_ on the surface of CuAg@C_Presod_. The sodiated interlayer enabled a high initial CE of 86% and a long lifespan exceeding 6500 h at 0.5 mA cm^−2^ current density. Surface measurements on pristine, sodiated, and desodiated CuAg@C_Presod_ reveal only minimal changes in the SEI. The similar elemental ratios in all three stages demonstrate that the artificial SEI reduces electrolyte decomposition and prevents Na dendrite formation during long‐term cycling.

## 4 Experimental Section

4.1

4.1.1

##### Synthesis of CuAg@C

First, a solution of 3.776 g PAN in 40 mL DMF was prepared, to which 2.815 g Cu(OAc)_2_ was added. This solution was stirred for one day at room temperature until a green solution was obtained. After one day, 2.633 g AgNO_3_ was added to the solution in a molar ratio of 1:1 with Cu(OAc)_2_, and the mixture was stirred for another day until a cyan‐colored solution was obtained. Electrospinning was performed using the solution (IME Medical Electrospinning, The Netherlands) under controlled climate conditions of 25 °C and 30% humidity. The solution was supplied to a syringe using an automatic pump at a rate of 20 μL min^−1^. The needle was moved parallel to the collector at a speed of 20 mm s^−1^ and a turn delay of 500 ns. The collector had a rotational speed of 700 rpm. Electrospinning was carried out for about 6.5 h until 8 mL of solution was consumed. The obtained fiber was then cross‐linked for 15 h at 250 °C with a heating rate of 5 °C per minute under oxygen. Afterward a reduction step for 2.5 h at 500 °C at the same heating rate under an Ar/H_2_ flow was applied to the fiber. For further tests, round discs with an area of 0.95 cm^2^ were punched out of the fiber.

##### Synthesis of CuAg@C_*Presod*_

For a presodiated sample, all synthesis steps were carried out under inert conditions in an Ar‐filled glovebox with water and oxygen contents below 0.1 ppm. To synthesize *n‐*BuNa, 1.44 g sodium tert‐butoxide was added to 6 mL of a 2.5 m *n‐*BuLi solution in hexane. The solution was stored inside a refrigerator at –40 °C. After two h, the solution was washed five times with 4 mL of hexane. The subsequent beige powder was dissolved in a mixture of 6 mL hexane and 6 mL THF. The *n‐*BuNa/hexane/THF solution was then used to presodiate CuAg@C. The obtained fiber disks of CuAg@C were immersed in 1 mL of *n‐*BuNa and stored for 1 h in a freezer at –40 °C. After that, the residual *n*‐BuNa solution was removed, and the soaked disk was heated at 300 °C for 30 min to obtain CuAg@C_Presod_.

##### Structural Characterizations

The Raman micro‐spectroscopy measurements were carried out using a WITec alpha300R (Oxford Instruments, UK) with a solid‐state 532 nm excitation laser with a laser power of 0.4 mW. The measurements were performed in an in‐operando ECC‐Opto‐STD cell (EL‐CELL, Germany) for ambient protection. The spectral parameters were determined by curve fitting using Origin (Originlab Corporation, United States) and followed the deconvolution method proposed by Brubaker et al. into five bands.^[^ ^25^ ^]^ A Pseudo‐Voigt function was used for the D1, D2, and G bands, while the D3 and D4 bands were fitted considering only Gaussian contributions.^[^ ^25^ ^]^

SEM was performed on an FEI Quanta FEG 650 (FEI/Thermo Fisher Scientific, USA) equipped with an energy dispersive X‐ray spectroscopy (EDAX)‐Octane 70 mm^2^ EDS detector (EDAX‐Ametek, USA). A K&W transfer module (Kammrath & Weiss, Germany) was used to transfer the samples from the glovebox directly to the SEM chamber without exposure to air.^[^ ^41^ ^]^ The SEM images were taken with an acceleration voltage of 2 kV and a spot size of 1 to ensure no material damage from the electron beam. For the SEM‐EDS map, the acceleration voltage was increased to 10 kV, and the spot size was increased to 6 nm.

STEM experiments were conducted on an FEI Titan G2 80‐200 (FEI/Thermo Fisher Scientific, USA) microscope equipped with a Cs‐probe corrector and an high‐angle annular dark‐field imaging detector. The microscope was used at 200 kV, and the semi‐angle of the probe was 24.7 mrad. The elemental distribution maps were obtained using EDS with four large‐solid‐angle symmetrical Si drift detectors.

XPS was performed on a K*α* spectrometer connected to a glovebox (Thermo Fisher Scientific, USA). The instrument operated at a 10^−9^ mbar base pressure with an equipped Al–K*α* X‐ray source. The surveys of the samples were measured with a pass energy of 200 eV and a spot size of 400 μm. The measurements of the elements were performed at the same spot, with the same spot size and a pass energy of 50 eV. A step size of 0.1 eV was applied. For depth profiling after the surface measurement was performed, a 2·1 mm spot was sputtered with Ar‐ions with an energy of 500 eV. Additional measurements were performed after 10, 500, 1041, and 2002 s of sputter time.

The XPS data were evaluated using the software Avantage (Thermo Fisher Scientific, USA). Quantification was performed using the instrument‐specific sensitivity factors obtained in Avantage. In addition, a correction factor dependent on kinetic energy (energy compensation factor, EC provided in Avantage) was applied to account for the inelastic scattering of photoelectrons as a function of depth in a homogeneous layer. A “smart background” was used to evaluate the core peaks. It was based on a Shirley‐type background with the additional restriction that the background was always less intense than the measured data. Voigt profiles with a 70% Gaussian and a 30% Lorentzian contribution were used for fitting. The cycled samples underwent the same cycling protocol as mentioned in the electrochemical measurements section and were stopped after the 5th cycle at 0.5 mAh.

##### Electrochemical Measurements

The plating/stripping and relevant impedance tests were performed in CR2032 coin cells with a Cu foil/sample‖electrolyte‖Na cell configuration. The commercial separator Celgard 2400 was chosen, along with 1 m NaPF_6_ in diglyme as the electrolyte. All the cells were tested using multichannel potentiostats (VMP3/MPG‐2, BioLogic, France) at 25 °C, controlled by a temperature chamber (Binder, Germany). The plating/stripping measurements were started with a formation plating step at 0.1 mA cm^−2^ for 25 h with consequent stripping at the same current until 0.4 V. Afterward, the current was switched to different densities with a fixed areal capacity of 2.5 mA h cm^−2^. Impedance measurements were performed using multichannel potentiostats (VMP3/MPG‐2, BioLogic, France) at 25 °C, within a frequency range of 100–100 mHz, with the allowed current of 50 μA. Direct current polarization measurements were performed in stainless steel||sample||stainless steel CR2032 coin cells without any electrolyte. A voltage of 2 V was applied to the cells for five minutes.

## Conflict of Interest

The authors declare no conflict of interest.

## Author Contributions

**Sandro Schöner**: conceptualization (lead); formal analysis (lead); investigation (lead); methodology (lead); visualization (lead); writing—original draft (lead). **Dana Schmidt**: formal analysis (supporting); writing—review & editing (supporting). **Leonie Wildersinn**: formal analysis (supporting); writing—review & editing (supporting). **Stephanie E. Wolf**: formal analysis (supporting); writing—review & editing (supporting). **Sebastian Speer**: investigation (supporting); writing—review & editing (supporting). **Beatrice Wolff**: investigation (supporting); writing—review & editing (supporting). **Arseniy Bokov**: investigation (supporting); writing—review & editing (supporting). **Pengfei Cao**: investigation (supporting); writing—review & editing (supporting). **Anna Windmüller**: writing—review & editing (supporting). **Xiaoxuan Chen**: investigation (supporting); writing—review & editing (supporting). **Chih‐Long Tsai**: writing—review & editing (supporting). **Fabian Jeschull**: formal analysis (supporting); supervision (supporting); writing—review & editing (supporting). **Hermann Tempel**: supervision (supporting); writing—review & editing (supporting). **Shicheng Yu**: conceptualization (supporting); formal analysis (supporting); project administration (lead); supervision (lead); visualization (supporting); writing—review & editing (lead). **Rüdiger‐A. Eichel**: funding acquisition (lead); resources (lead); supervision (supporting); writing—review & editing (supporting).

## Supporting information

Supplementary Material

## Data Availability

The data that support the findings of this study are available from the corresponding author upon reasonable request.
