# Supplementary material for: Stable Na Deposition/Dissolution Enabled by 3D Bimetallic Carbon Fibers with Artificial Solid Electrolyte Interface
Source: Small Sci. 2025 Apr 29;5(6):2400655. doi: 10.1002/smsc.202400655 (PMC12168592; doi:10.1002/smsc.202400655)
Supplement: Supplementary file 1 — Supplementary Material [file SMSC-5-2400655-s001.pdf]

Supporting Information

Stable Na Deposition/Dissolution Enabled by Three-Dimensional Bimetallic Carbon Fibers with Artificial SEI

Sandro Schöner[1,2], Dana Schmidt[1,2], Leonie Wildersinn[3], Stephanie E. Wolf[1,2], Sebastian Speer[1,2], Beatrice Wolff[1,2] Arseniy Bokov[3,4], Pengfei Cao[5], Anna Windmüller[1], Xiaoxuan Chen[1], Chih-Long Tsai[1], Fabian Jeschull[3], Hermann Tempel[1], Shicheng Yu[1] and Rüdiger-A. Eichel[1,2,6]*

[1]S. Schöner, D. Schmidt, S. E. Wolf, S. Speer, B. Wolff, A. Windmüller, X. Chen, C. Tsai, H. Tempel, S. Yu, R.-A. Eichel
Institute of Energy Technologies – Fundamental Electrochemistry (IET-1),
Forschungszentrum Jülich, 52428 Jülich, Germany
E-mail: s.yu@fz-juelich.de

[2]S. Schöner, D. Schmidt, S. E. Wolf, S. Speer, B.Wolff, R.-A. Eichel
Material and Processes of Electrochemical Energy Storage and Conversion,
RWTH Aachen University, 52074 Aachen, Germany

[3]L. Wildersinn, A. Bokov, F. Jeschull
Karlsruher Institute of Technologie (KIT), Institute for Applied Materials-Energy Storage Systems (IAM-ESS), 76344 Eggenstein Leopoldshafen, Germany

[4]A. Bokov
Helmholtz Institute Ulm (HIU), Electrochemical Energy Storage (EES),
89081 Ulm, Germany

[5]P. Cao
Ernst Ruska-Centre for Microscopy and Spectroscopy with Electrons,
Forschungszentrum Jülich, 52428 Jülich, Germany

[6]R.-A. Eichel
Institute of Energy Materials and Devices - Helmholtz Institute Münster: Ionics in Energy
Storage (IMD-4 / HI MS)
Forschungszentrum Jülich, 48149 Münster, Germany

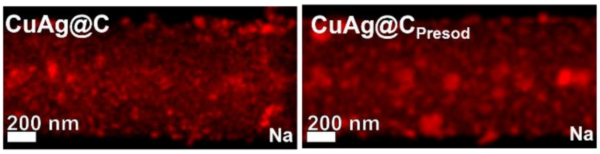

Figure S1 STEM-EDS maps of CuAg@C and CuAg@C_{Presod} showing the element distribution for Na.

Table S1 STEM-EDS quantification results of CuAg@C and CuAg@C$_{Presod}$.

	Element	Atomic fraction [%}	Atomic error [%]	Mass fraction [%]	Mass error [%]	Fit error [%}
**CuAg@C**	C	65.79	2.42	33.28	1.89	3.08
	N	15.31	2.71	9.03	1.76	5.23
	O	3.49	0.70	2.35	0.48	3.65
	Na	0.34	0.07	0.33	0.07	2.21
	Cu	7.19	1.00	19.25	2.40	0.19
	Ag	7.87	0.89	35.76	2.91	0.06
	**Element**	**Atomic fraction [%}**	**Atomic error [%]**	**Mass fraction [%]**	**Mass error [%]**	**Fit error [%}**
**CuAg@C$_{Presod}$**	C	55.89	2.21	28.24	1.57	2.81
	N	9.45	1.79	5.57	1.12	4.75
	O	10.47	1.91	7.05	1.36	1.03
	Na	10.12	1.85	9.79	1.83	0.18
	Cu	7.76	1.07	20.75	2.52	0.34
	Ag	6.30	0.72	28.60	2.61	0.14

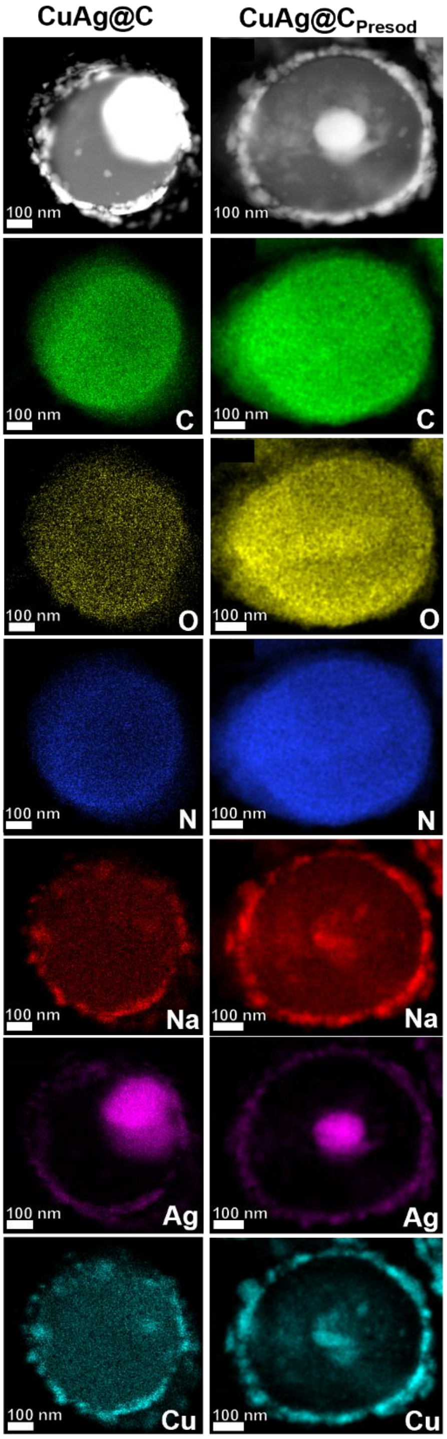

Figure S2 STEM-EDS maps showing the field of view and the elemental distribution for C, O, N, Na, Ag and Cu for CuAg@C (left) and CuAg@C_{Presod} (right).

Table S2 Spectral parameters for the first-order Raman bands of CuAg@C and $CuAg@C_{Presod}$, showing the band position (Stokes Raman shift), full width at half maximum (FWHM), the peak area (A) and the peak area ratios.

**Band**	**Parameter**	**CuAg@C**	**$CuAg@C_{Presod}$**
**G**	Position [cm^{-1}]	1,590	1,551
	FWHM [cm^{-1}]	99.73	86.59
	A [cm^{2}]	52	52
**D1**	Position [cm^{-1}]	1,350	1,343
	FWHM [cm^{-1}]	142.08	132
	A [cm^{2}]	189	162
	A_{D1}/A_{G}	3.66	3.09
**D2**	Position [cm^{-1}]	1,625	1,599
	FWHM [cm^{-1}]	50	55.24
	A [cm^{2}]	5	12
**D3**	Position [cm^{-1}]	1,504	1,459
	FWHM [cm^{-1}]	147	147
	A [cm^{2}]	39	56
	A_{D3+D4}/A_{total}	0.21	0.28
**D4**	Position [cm^{-1}]	1,200	1,221
	FWHM [cm^{-1}]	173.72	150
	A [cm^{2}]	26	30
	A_{D4+D3}/A_{total}	0.21	0.28

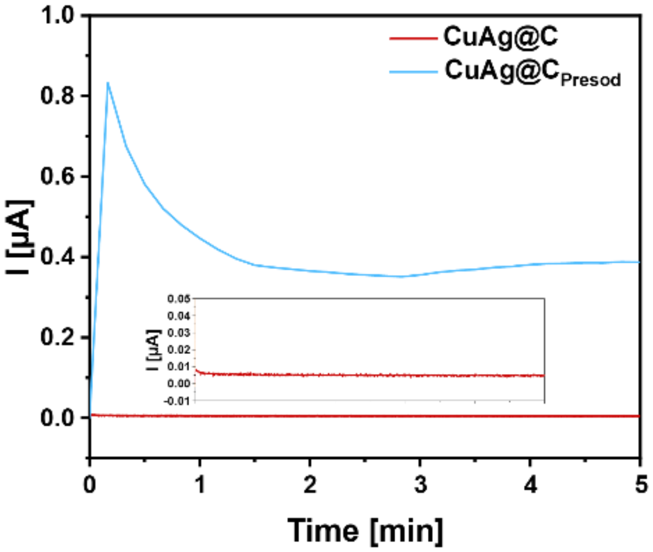

Figure S3 Current vs. time curves of CuAg@C and CuAg@C_{Presod} measured in R2032 coin cells. A symmetric cell setup containing stainless steel||sample||stainless steel was constructed without any electrolyte. The current vs. time curves were recorded at 2 V for five minutes at 25 ºC.

Table S3 Measurement details for the different XPS scans.

ID	Scans	Dwell time [ms]	Pass energy [eV]	Range [eV]
Survey	10	10	200	10-1350
C 1s	5	50	50	279-298
O 1s	5	50	50	520-545
Na 1s	10	50	50	1062-1085
N 1s	10	50	50	392-410
Cu 2p	3	50	50	925-965
Ag 3d	10	50	50	360-380
P 2p	10	50	50	124-144
F 1s	10	50	50	678-698

Table S4 Quantification results and fitting parameters of different components of CuAg@C. Quantification refers to boldly written elements.

Component	**C** (CNF)	**C**N (**C**-O)	**C**=O	**N** (pyridinic)	-**N**H_2	**N** (pyrolic)	**N** (graphitc[1])	**N** (graphitc[2]	C-**O**	C=**O**	**Ag**	**Cu**
**Surface [%]**	40.8	15.43	2.02	6.2	9.77	1.48	2.83	1.48	1.41	3.02	9.93	5.62
**Surface BE [eV]**	284.61	286.25	288.02	398	398.73	399.7	400.4	401.5	532.52	530.62	368.15	932.44
**Surface FWHM [eV]**	1.34	1.57	2	1.3	1.32	1.42	1.3	1.6	1.86	1.76	0.81	1.53
**Surface Intensity [CPS]**	23094.22	11244.34	2358.07	4556.34	11165.63	2430.3	1974.49	1917.98	1766.73	4021	146409.7	35165.33
**10 s sputter time [%]**	45.89	13.04	1.36	3.22	6.82	2.11	2.73	1.32	0.61	1.07	13.39	8.43
**10 s sputter time BE [eV]**	284.63	286.13	288.12	398	398.75	399.7	400.4	401.5	532.47	530.67	368.14	932.61
**10 s sputter time FWHM [eV]**	1.6	1.8	2	1.36	1.37	1.57	1.35	1.66	1.77	1.82	0.88	1.64
**10 s sputter time Intensity [CPS]**	20499.97	8041.53	2064.26	2255.98	7061.32	1904.2	2173.52	963.16	760.47	1304.06	161100.55	42157.71
**500 s sputter time [%]**	49.1	16.31	2.81	4.59	4.76	2.05	2.37	1.32			7.08	9.6
**500 s sputter time BE [eV]**	284.49	285.93	288.12	398	398.75	399.7	400.49	401.5			368.17	932.72
**500 s sputter time FWHM [eV]**	1.6	1.8	2	1.47	1.31	1.6	1.3	1.6			0.88	1.71
**500 s sputter time Intensity [CPS]**	25453.28	10187.17	2725.9	3253.07	5649.4	2836.04	1308.36	1631.47			92209.16	49270.82
**1041 s sputter time [%]**	53.28	15.99	2.69	4.92	4.47	1.47	2.92	0.85			5.57	7.84
**1041 s sputter time BE [eV]**	284.48	285.93	288.12	398	398.75	399.7	400.4	401.5			368.15	932.76
**1041 s sputter time FWHM [eV]**	1.57	1.8	2	1.6	1.31	1.57	1.57	1.6			0.88	1.75
**1041 s sputter time Intensity [CPS]**	29173.98	11050.01	2780.16	3530.66	5203.8	2435.17	1797.61	1362.64			74676.14	41161.49
**2002 s sputter time[%]**	56.02	16.28	2.32	4.86	3.99	1.91	2.53	0.61			4.62	6.87
**2002 s sputter time BE [eV]**	284.45	285.85	288.02	398	398.75	399.7	400.4	401.4			368.17	932.79
**2002 s sputter time FWHM [eV]**	1.56	1.79	2	1.53	1.31	1.6	1.3	1.6			0.89	1.81
**2002 s sputter time Intensity [CPS]**	30767.72	11601.98	2916.97	3706.54	4678.3	2784.88	1834.99	969.38			62756	36100.89

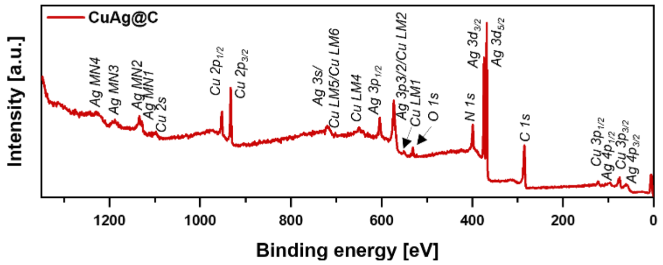

Figure S4 Survey measurement of the XPS spectrum for CuAg@C.

Table S5 Quantification results of different components of CuAg@C_{Presod}. Quantification refers to boldly written elements.

Component	**C** (CNF)	**C**-C/**C**-H	R**C**ONa	R**C**O$_2$Na	Na$_2$**C**O$_3$	**N**^{1}	**N**^{2}	C-**O**	C=**O**	Na$_2$**O**	**Ag**	**Ag**Na	**Cu**	**Na**
**Surface [%]**	2.5	42.75	0.79	4.01	4.44			0.57	25.83			0.1		11.82
**Surface BE [eV]**	283.63	284.8	286.22	288.22	289.23			532.99	530.96			368.24		1071.07
**Surface FWHM [eV]**	1.3	1.22	1.3	1.3	1.3			1.32	1.52			0.76		1.53
**Surface Intensity [CPS]**	2042.64	37172.89	641.5	3262.37	3606.84			1101.12	43478.53			20601.11		39790.46
**10 s sputter time [%]**	25.36	13.63	1.86	1.89	6.08	1.77		0.57	25.54	2.16	1.61	0.1	3.27	16.16
**10 s sputter time BE [eV]**	284	284.8	286.19	288.19	289.3	398.25		533.05	530.98	529.21	367.38	367.98	932.15	1071.21
**10 s sputter time FWHM [eV]**	1.54	1.21	1.46	1.6	1.6	3.5		1.15	1.74	1.69	0.72	0.9	1.63	1.67
**10 s sputter time Intensity [CPS]**	19690.03	13400.18	1521.95	1401.87	4516.87	942.73		1431.67	42366.77	3698.91	28761.24	1457.03	20269.28	56388.57
**500 s sputter time [%]**	33.07	11.42	3.85	2.29	3.81	4.89	1.48	0.42	14.67	0.72	2.79	0.73	7.4	12.47
**500 s sputter time BE [eV]**	283.57	284.8	286.27	288.17	289.28	397.71	399.77	533.1	530.87	529.06	367.16	367.79	931.84	1071.25
**500 s sputter time FWHM [eV]**	1.6	1.49	1.6	1.6	1.6	1.71	2	1.59	2	1.51	0.79	0.9	1.73	1.72
**500 s sputter time Intensity [CPS]**	24932.74	9219.18	2894.22	1718.83	2863.68	5336.21	1383.28	771.55	21380.14	1385.63	45937.45	10461.77	45803.22	42673.56
**1041 s sputter time [%]**	36.61	13.09	2.49	3.31	4.06	7.17	1.2	0.81	10.08	0.85	2.33	0.57	5.95	11.48
**1041 s sputter time BE [eV]**	283.46	284.8	286.17	288.17	289.28	397.64	399.58	532.8	530.81	529.29	367.06	367.7	931.8	1071.36
**1041 s sputter time FWHM [eV]**	1.6	1.53	1.6	1.6	1.6	1.97	2	2	1.92	1.37	0.76	0.9	1.85	1.72
**1041 s sputter time Intensity [CPS]**	28198.51	10534.17	3123.07	1908.39	2539.02	6948.69	1148.58	1205.72	15667.79	1851.74	39987.56	8277.85	35935.91	40051.16
**2002 s sputter time[%]**	40.24	12.88	5.21	2.4	3.07	7.13	1.86	0.71	7.25	0.96	2.05	0.39	5.56	10.3
**2002 s sputter time BE [eV]**	283.47	284.8	286.22	288.17	289.28	397.65	399.63	532.96	530.8	529.36	367.1	367.8	931.93	1071.37
**2002 s sputter time FWHM [eV]**	1.6	1.42	1.6	1.6	1.6	1.79	2	2	1.93	2	0.78	0.9	1.94	1.69
**2002 s sputter time Intensity [CPS]**	30995.17	11182.43	4003.96	1839.23	2355.93	7609.53	1776.22	1048.08	11202.63	1423.74	34932.71	5664.44	32164.28	36540.88

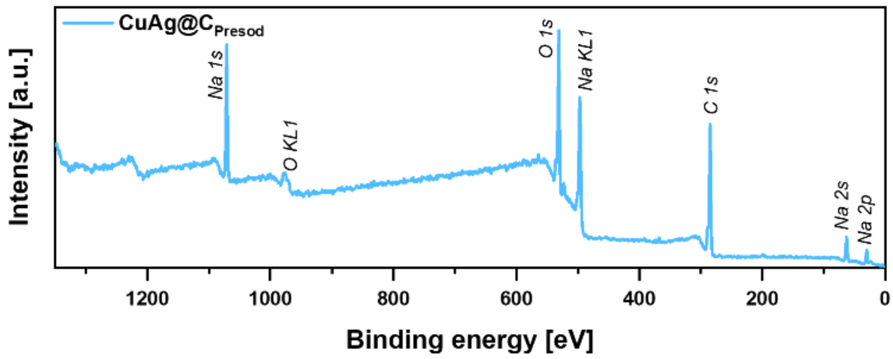

Figure S5 Survey measurement of the XPS spectrum for CuAg@C_{Presod}.

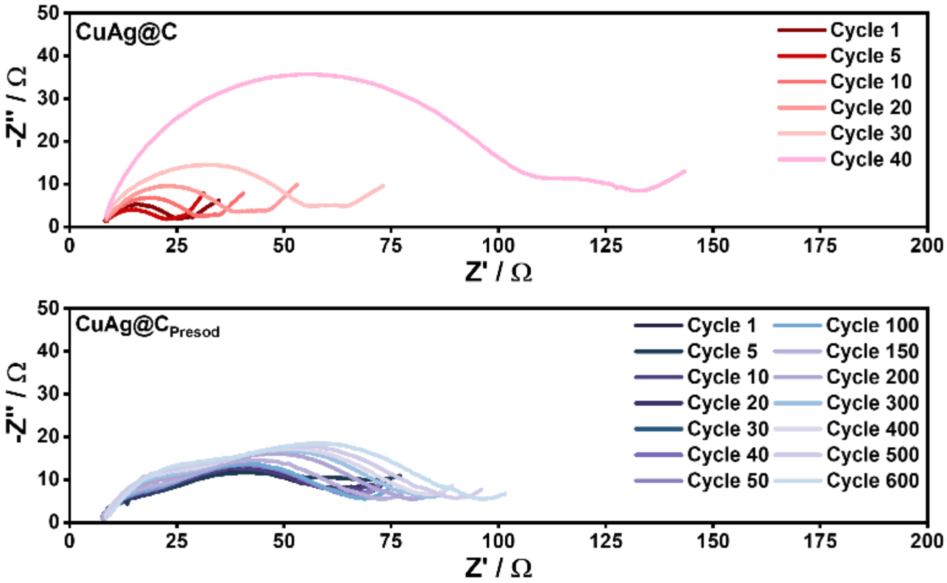

Figure S6 Impedance measurements for g) CuAg@C and CuAg@C$_{Presod}$ after certain cycle numbers. All samples were tested in a Cu foil/sample||electrolyte||Na cell setup.

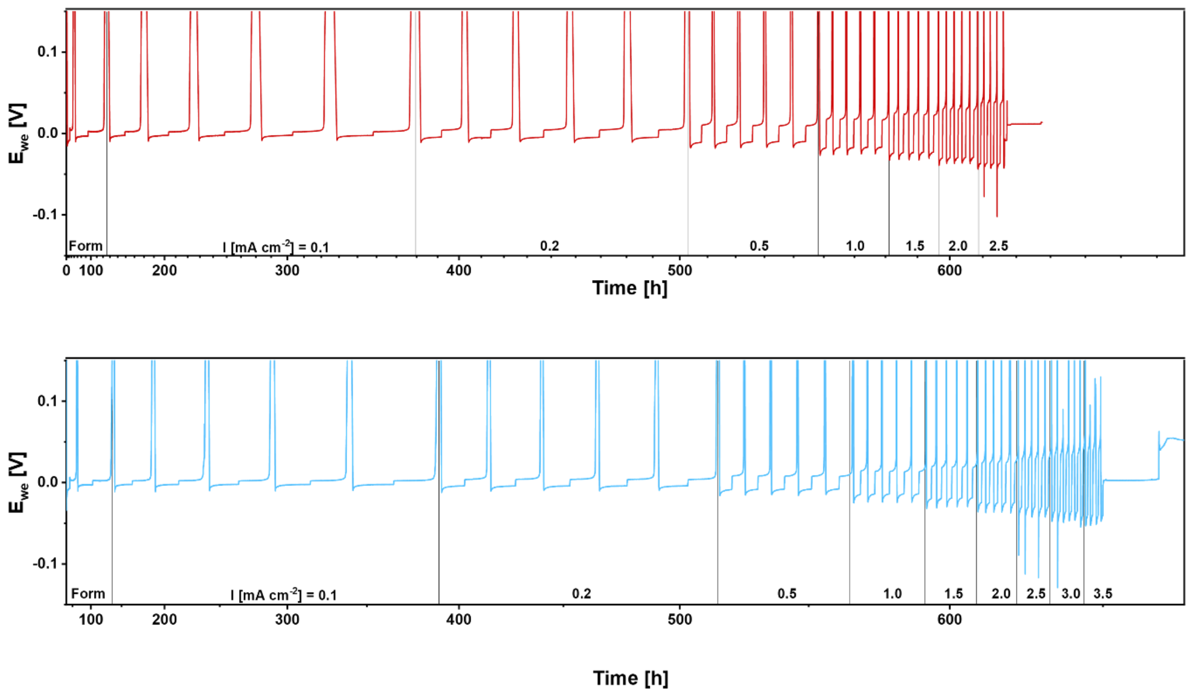

Figure S7 Rate performance of CuAg@C and CuAg@C$_{Presod.}$ The samples were tested in a Cu foil/sample||electrolyte||Na cell setup at room temperature.

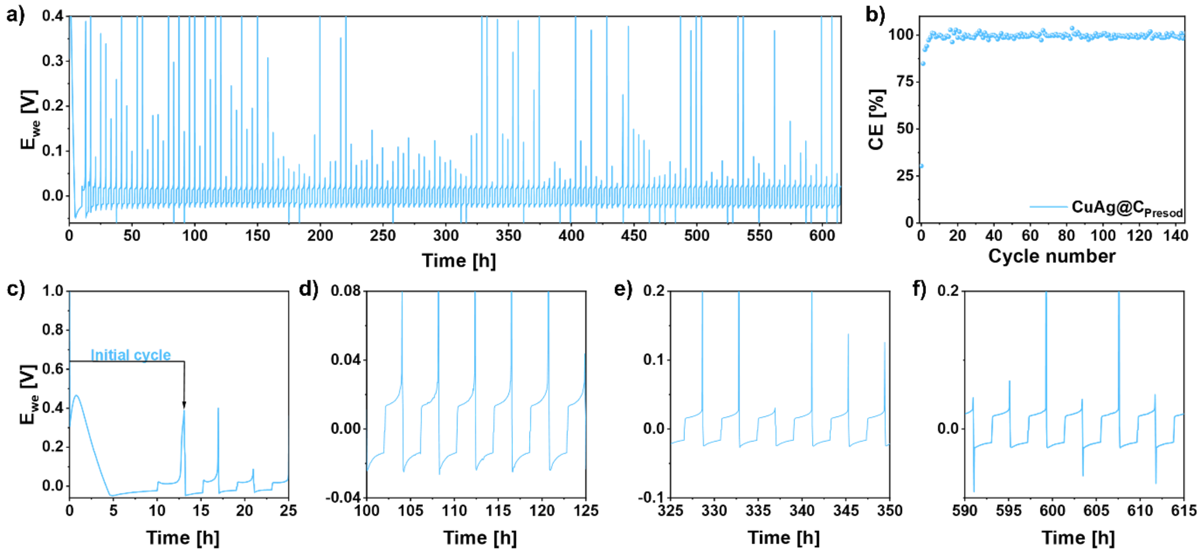

Figure S8 a) Voltage vs. time profile of CuAg@C$_{Presod}$ during Na plating/stripping tests. b) Relevant Coulombic efficiencies for the voltage vs. time profile depicted in a). c)-f) Zoomed-in sections from a) at different periods

Table S6 Spectral parameters for the first-order Raman bands of CuAg@C_desodiated and CuAg@C_sodiated, showing the band position (Stokes Raman shift), full width at half maximum (FWHM), the peak area (A) and the peak area ratios.

Band	Parameter	CuAg@C_desodiated	CuAg@C_sodiated
**G**	Position [cm^{-1}]	1,586	1,577
	FWHM [cm^{-1}]	76.32	76.59
	A [cm^2]	42	54
**D1**	Position [cm^{-1}]	1,337	1,335
	FWHM [cm^{-1}]	113.68	140.23
	A [cm^2]	153	44
	A_{D1}/A_G	3.64	2.68
**D2**	Position [cm^{-1}]	1,615	1,625
	FWHM [cm^{-1}]	35.66	0.01
	A [cm^2]	5	0
**D3**	Position [cm^{-1}]	1,500	1,492
	FWHM [cm^{-1}]	151.27	145.03
	A [cm^2]	71	97
	A_{D3+D4}/A_{total}	0.30	0.36
**D4**	Position [cm^{-1}]	1,200	1,200
	FWHM [cm^{-1}]	339.49	95.91
	A [cm^2]	16	12
	A_{D4+D3}/A_{total}	0.30	0.36

Table S7 Spectral parameters for the first-order Raman bands of CuAg@C$_{Presod}$_desodiated and CuAg@C$_{Presod}$_sodiated, showing the band position (Stokes Raman shift), full width at half maximum (FWHM), the peak area (A) and the peak area ratios.

**Band**	**Parameter**	**CuAg@C$_{Presod}$_desodiated**	**CuAg@C'$_{Presod}$_sodiated**
**G**	Position [cm^1]	1,586	1,570
	FWHM [cm^{-1}]	76.32	72.41
	A [cm^2]	42	26
**D1**	Position [cm^1]	1,337	1,335
	FWHM [cm^{-1}]	113.68	67.05
	A [cm^2]	153	44
	A_{D1}/A_G	2.9	1.74
**D2**	Position [cm^1]	1,615	1,615
	FWHM [cm^{-1}]	35.66	0
	A [cm^2]	5	0
**D3**	Position [cm^1]	1,500	1,500
	FWHM [cm^{-1}]	151.27	156.45
	A [cm^2]	71	134
	A_{D3+D4}/A_{total}	0.28	0.67
**D4**	Postion [cm^{-1}]	1,200	1,270
	FWHM [cm^{-1}]	153	50
	A [cm^2]	8	11
	A_{D4+D3}/A_{total}	0.28	0.67

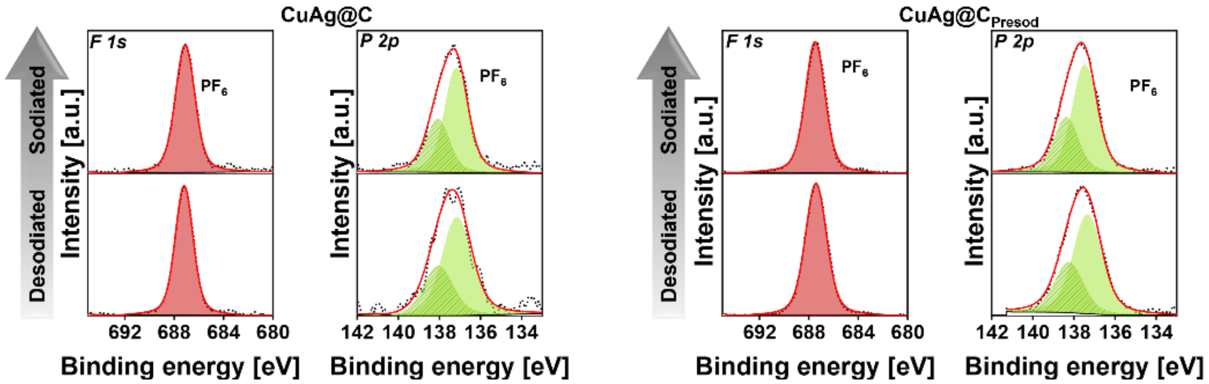

Figure S9 *F 1s* and *P 2p* XPS spectra for CuAg@C and CuAg@C$_{Presod}$.in a sodiated (top) and desodiated (bottom) state after five cycles using the same protocol as for the stability tests. For comparison, the measurements are referenced to 284.8 eV for the signal referring to C-C/C-H. Detailed measurement and fitting parameters can be found in **Table S8**. Survey measurements for all four samples can be found in **Figure S8** and **Figure S9**. All spectra are normalized, with the highest signal in each spectrum is set to 1.

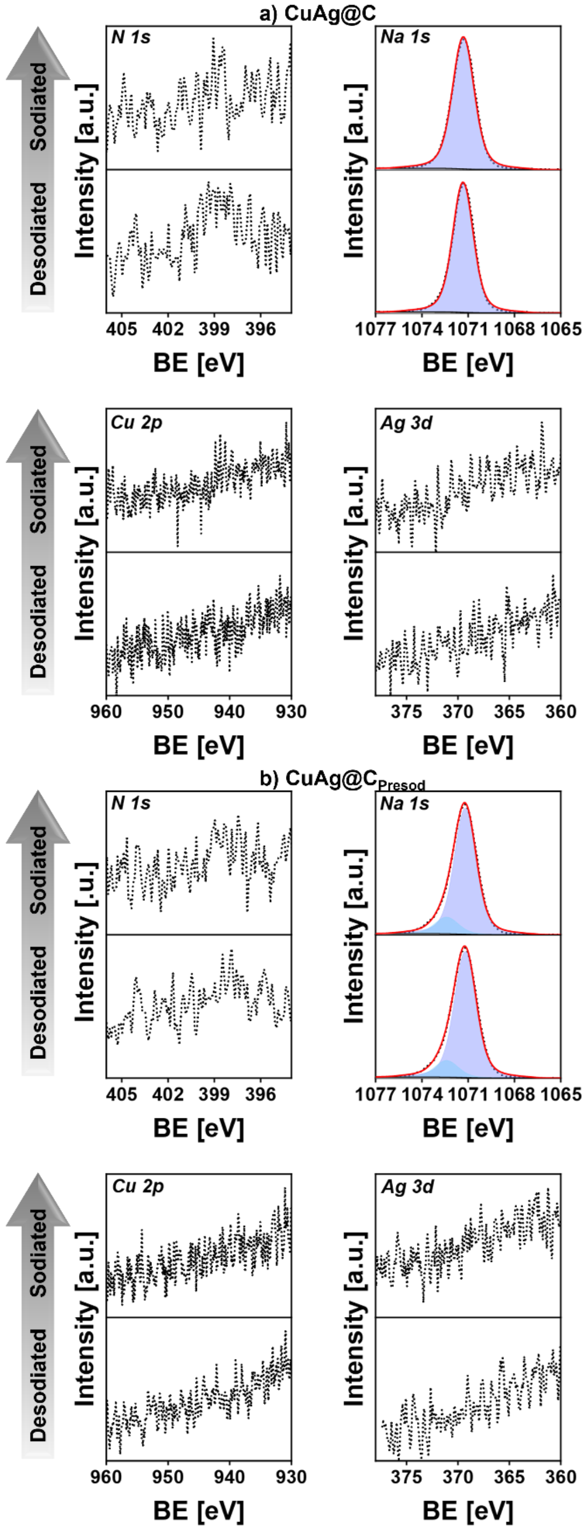

Figure S10 *N 1s*, *Na 1s*, *Cu 2p* and *Ag 3d* XPS spectra for a) CuAg@C and b) CuAg@C$_{Presod}$.in a sodiated (top) and desodiated (bottom) state after five cycles using the same protocol as for the stability tests. For comparison, the measurements are referenced to 284.8 eV for the signal referring to C-C/C-H. Detailed measurement and fitting parameters can be found in **Table S8**. Survey measurements for all four samples can be found in **Figure S8** and **Figure S9**. All spectra are normalized, with the highest signal in each spectrum is set to 1.

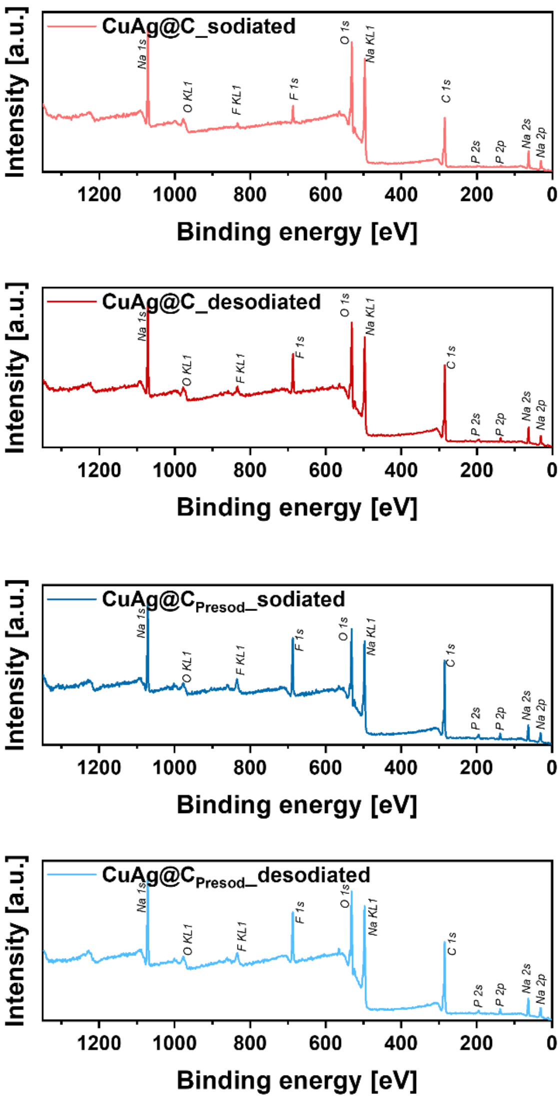

Figure S11 Survey measurement of the XPS spectrum for CuAg@C_sodiated, CuAg@C_desodiated, CuAg@C$_{Presod}$_sodiated (top) and CuAg@C$_{Presod}$_desodiated (bottom).

Table S8 Quantification results and fitting parameters of different cycled materials. Quantification refers to boldly written elements.

**Component**	**C**-C/**C**-H	R**C**ONa	R**C**O$_2$Na	Na$_2$**C**O$_3$	C-**O**	C=**O**	**Na**^{1}	**Na**^{2}	P**F**$_x$	**P**F$_x$
**CuAg@C_desodiated [%]**	39.65	3.28	4.52	4.56	1.32	22.84	13.6		8.67	1.57
**CuAg@C_desodiated BE [eV]**	284.8	286.53	288.53	289.24	532.91	531.19	1071.34		687.2	137.18
**CuAg@C_desodiated FWHM [eV]**	1.25	1.3	1.3	1.3	1.6	1.5	1.58		1.72	1.35
**CuAg@C_desodiated Intensity [CPS]**	46694.21	3721.09	5114.14	5160.48	2934.31	54469.67	61800.25		22528.32	1685.3
**CuAg@C_sodiated [%]**	30.15	3.77	4.11	6.97	2.22	28.57	18.05		5.11	1.04
**CuAg@C_sodiated BE [eV]**	284.8	286.45	288.43	289.23	532.65	531.19	1071.32		687.12	137.16
**CuAg@C_sodiated FWHM [eV]**	1.3	1.6	1.3	1.3	1.6	1.59	1.67		1.87	1.73
**CuAg@C_sodiated Intensity [CPS]**	30382.86	3076.27	4119.29	6989.01	4379	56840.15	68787.07		10776.17	138.06
**CuAg@C$_{Presod}$_desodiated [%]**	38.4	1.32	4.6	4.14	0.4	20.98	12.04	1.72	12.55	2.13
**CuAg@C$_{Presod}$_desodiated BE [eV]**	284.8	286.7	288.52	289.43	532.46	531.16	1071.19	1072.4	687.42	137.34
**CuAg@C$_{Presod}$_desodiated FWHM [eV]**	1.49	1.6	1.53	1.42	1.05	1.72	1.7	1.82	2.05	1.65
**CuAg@C$_{Presod}$_desodiated Intensity [CPS]**	38465.41	1233.18	4495.21	4347.1	1509.84	47539.77	51629.81	6858.91	27738.47	1891.54
**CuAg@C$_{Presod}$_sodiated [%]**	37.78	2.57	4.77	3.34	0.69	20.34	11.24	2.35	14.31	2.61
**CuAg@C$_{Presod}$_sodiated BE [eV]**	284.8	286.5	288.52	289.27	532.95	531.34	1071.4	1072.48	687.49	137.47
**CuAg@C$_{Presod}$_sodiated FWHM [eV]**	1.22	1.3	1.3	1.3	1.37	1.63	1.51	1.97	1.9	1.42
**CuAg@C$_{Presod}$_sodiated Intensity [CPS]**	43523.02	2774.76	5139.77	3598.39	1697.42	42341.13	51120.59	8111.66	31964.1	2522.76

Table S9 Overview of different modifications for the Na metal deposition/dissolution behavior.

Reference	System (Current Collector Modificatrion)	Cycling life	CE[%]	capacity
**1**	Zn@Ag‖Na	500 Cycles	99.9	1.0 mA h cm^{-2}
**2**	Na/NaSn‖Na	400 Cycles	n.a.	4.0 mA h cm^{-2}
**3**	NGAL-Na‖ NGAL-Na	214	n.a.	3.0 mA h cm^{-2}
**4**	Cu@Au‖Na	300	99.8	1.0 mA h cm^{-2}
	**System (Interlayer Modification)**			
**5**	Co_3O_4@C/rGO‖Na	395 Cycles	99.87	1.0 mA h cm^{-2}
**6**	HrGF‖Na	150 Cycles	98	1.0 mA h cm^{-2}
**7**	F-CTN‖F-CTN	600	99.5	1.0 mA h cm^{-2}
**8**	cyano-sp^2c-COF‖Na	500	99.7%	1.0 mA h cm^{-2}

References

[1] X. Chen, X. Zhou, Z. Yang, Z. Hao, J. Chen, W. Kuang, X. Shi, X. Wu, L. Li, S.-L. Chou, *Chem. Sci.*, **2024**, *15*, 4833

[2] Q. Jin, H. Lu, Z. Zhang, J. Xu, B. Sun, Y. Jin, K. Jiang, *Adc. Sci.*, **2022**, *9*, *7*, 2103845.

[3] X. Lv, F. Tang, Y. Yao, C. Xu, D. Chen, L. Liu, Y. Feng, X. Rui, Y. Yu, *Susmat*, ***2022***, *2*, *6*, 699.

[4] S. Tang, Z. Qiu, X.-Y. Wang, Y. Gu, X.-G. Zhang, W.-W. Wang, J.-W. YXan, M.-S. Zheng, Q.-F. Dong, B.-W. Mao, *Nano Energy*, **2018**, *48*, 101.

[5] W. Bai, H. Wang, D. H. Min, J. Miao, B. Li, T. Xu, D. Kong, X. Li, X. Yu, Y. Wang, H. S. Park, *Adv. Sci.* , **2024**, *11*, *35*, 2404419.

[6] S. J. Lee, D. Kang, D. Y. Hyeon, D. S. Kim, S. Eom, S. H. Jeong, D. P. Lee, D. Baek, J.-H. Ahm, G. H. Ryu, K.-I. Park, S. Moon, J.-H. Kim, *Energy Storage Mater.*, **2024**, *64*, 103047.

[7] R. Zhuang, X. Cai, C. Qu, S. He, S. Kaskel, A. Y. Shenouda, H. Wang, F. Xu, *Carbon*, **2024**, *221*, 118862.

[8] R. Zhuang, C. Qu, J. Yang, S. Xu, F. Xu, *J. Polym. Sci.*, **2024**, *62*, *21*, 4898.
